# Variation of virulence of five *Aspergillus fumigatus* isolates in four different infection models

**DOI:** 10.1371/journal.pone.0252948

**Published:** 2021-07-09

**Authors:** E. M. Keizer, I. D. Valdes, G. Forn-Cuni, E. Klijn, A. H. Meijer, F. Hillman, H. A. B. Wösten, H. de Cock

**Affiliations:** 1 Microbiology, Department of Biology, Utrecht University, Utrecht, The Netherlands; 2 Institute of Biomembranes, Utrecht University, Utrecht, The Netherlands; 3 Institute of Biology Leiden, Leiden University, Leiden, The Netherlands; 4 Junior Research Group Evolution of Microbial Interactions, Leibniz Institute for Natural Product Research and Infection Biology, Hans Knöll Institute (HKI), Jena, Germany; Dartmouth College, Geisel School of Medicine, UNITED STATES

## Abstract

Conidia of *Aspergillus fumigatus* are inhaled by humans on daily basis. As a consequence, these conidia can cause infections that differ in severity ranging from allergic bronchopulmonary aspergillosis to invasive aspergillosis. In this study we compared virulence of five *A*. *fumigatus* isolates in four different infection models to address the predictive value of different model systems. Two of the *A*. *fumigatus* strains were isolated from dogs with a non-invasive sino-nasal aspergillosis (DTO271-B5 and DTO303-F3), while three strains were isolated from human patients with invasive aspergillosis (Af293, ATCC46645 and CEA10). Infection models used encompassed cultured type II A549 lung epithelial cells, *Protostelium aurantium* amoeba, *Galleria melonella* larvae and zebrafish embryos. No major differences in virulence between these five strains were observed in the lung epithelial cell model. In contrast, strain ATCC46645 was most virulent in the amoeba and zebrafish model, whereas it was much less virulent in the *Galleria* infection model. DTO303-F3 was most virulent in the latter model. In general, reference strain Af293 was less virulent as compared to the other strains. Genome sequence analysis showed that this latter strain differed from the other four strains in 136 SNPs in virulence-related genes. Together, our results show that virulence of individual *A*. *fumigatus* strains show significant differences between infection models. We conclude that the predictive value of different model systems varies since the relative virulence across fungal strains does not hold up across different infection model systems.

## Introduction

*Aspergillus fumigatus* is a saprotrophic fungus, feeding on dead or living organic matter [1]. The 2–3 μm wide conidia [2] are dispersed via the air and hundreds of these conidia are inhaled by humans daily [3]. Upon inhalation, these conidia can cause lung infections that differ in severity. Allergic bronchopulmonary aspergillosis (ABPA), chronic pulmonary aspergillosis (CPA) and *Aspergillus* bronchitis are classified as non-invasive aspergillosis infections that can occur in immune-competent patients. ABPA is the result from sensitization to *A*. *fumigatus* allergens triggering an allergic inflammatory response in the bronchial airways [4]. For instance, it occurs in about 2.5% of all patients with asthma and in 1–15% of patients with cystic fibrosis [5,6]. CPA manifests as an aspergilloma, a chronic cavity pulmonary aspergillosis, or as a chronic fibrosing pulmonary aspergillosis [7]. Less than 1% of asthma patients and around 5% of cystic fibrosis patients develop CPA next to ABPA [5,8]. Finally, *Aspergillus* bronchitis is characterized by a superficial, but chronic infection of the airways. It is for instance found in a small proportion (1.6%) of CF patients [9]. Next to lung infections, *A*. *fumigatus* is also a causative agent of non-invasive infections in sino-nasal areas in humans and animals [10]. In dogs, *A*. *fumigatus* is the most commonly identified fungal species in sino-nasal aspergillosis (SNA) [11].

The most severe infection caused by *A*. *fumigatus* is an invasive pulmonary aspergillosis (IPA). IPA develops especially in immune-compromised patients. Hyphae resulting from inhaled conidia invade the respiratory mucosa, which can be followed by invasion of other organs [12]. Stem cell recipients are the highest risk group for these infection [13], followed by solid organ transplant recipients [14]. The high risk is caused by the severe immune suppression caused by medication that is needed for these transplantations [15].

Genome-wide comparative analysis and single nucleotide polymorphism (SNP) calling revealed that 101 *A*. *fumigatus* isolates from clinical and environmental origin were distributed in four clusters. 0.3% and 0.2% of the reference genomes of the clinical strains Af293 and A1163 (CEA10) was identified as a SNP, respectively [16]. Significant genetic and phenotypic variability related to pathogenicity and stress tolerance was observed between 16 clinical *A*. *fumigatus* isolates [17]. However, no correlation was seen between the observed genetic and phenotypic variabilities and disease phenotype in humans. A comparison between the clinical isolates Af293 and CEA10 revealed that the former is more susceptible to hypoxic conditions than the latter [18]. Interestingly, this study also revealed a correlation between reduced fitness at low oxygen and reduced virulence in an immune-suppressed murine IPA model. Virulence of Af293 and CEA10 was also compared in a zebrafish infection model. These studies showed that the faster growing CEA10 strain is more virulent than the slower growing Af293 strain [19]. Together, differences have been observed between virulence of different *A*. *fumigatus* strains. Yet, no extensive systematic comparisons have between in infection models.

In this study we compared the relative virulence of five *A*. *fumigatus* isolates in four different infection models to address the predictive value of different model systems. We used five *A*. *fumigatus* isolates that originated from invasive and non-invasive infections and related their virulence to SNPs. To this end, A549 type II lung epithelial lung cells, amoebae, larvae of *Galleria melonella*, and zebrafish embryos were used as models of infection. Type II A549 lung epithelial cells have been used extensively to study the lifestyle of *Aspergillus* species but in most studies only a limited set of strains were compared [20–22]. In the study of Wasylnka & Moore, 2002 [23] this system revealed no major differences in binding, uptake and germination between *A*. *fumigatus* strains ATCC13073 and CHUV. In addition, expression analysis of Af293 and CEA10 during infection of A549 cells did not reveal a similar response of the fungus. It was proposed that these differences were due to the different backgrounds of the strains and not the presence of the A549 cells [24].

Previous research showed that amoeba and innate immune cells can trigger similar responses to fungi such as *Cryptococcus neoformans* and partially also to *A*. *fumigatus* [25]. Upon interaction with the model amoeba *Dictyostelium discoideum*, conidia are internalized with a similar efficiency as during phagocytosis by human monocytes [26]. Fungivorous amoeba such as *Protostelium aurantium* can feed on conidia of *A*. *fumigatus* [27]. This suggests that amoebae can be used as an early evolutionary model for human immune cells.

The innate immune system is also the main defence against microbial pathogens in insects such as *Galleria melonella*. Due to this similarity, larvae of *G*. *melonella* can be used as an infection model. The haemolymph of the larvae contains anti-microbial peptides and haemocytes, similar to human phagocytes, to clear microbial pathogens [28]. A correlation between gliotoxin production and virulence of *A*. *fumigatus* isolates was suggested in this model [29]. In addition, *A*. *fumigatus* siderophore mutants show strongly reduced virulence in *G*. *melonella* larvae and a high correlation with survival of mice upon infection [30]. Similar results were observed with mutants of the dimorphic pathogen *Candida albicans* [31]. Comparison of three wild-type *A*. *fumigatus* strains in *G*. *melonella* larvae showed differences in survival of the larvae. Conidia of ATCC46645 were most virulent, while conidia of D141 and Af237 had a similar virulence [30].

Transparent zebrafish embryos are also a good model to study microbial infections. The hindbrain of the embryos consists of an epithelial cell layer into which the conidia of *A*. *fumigatus* can be injected [32]. The zebrafish embryo has a functional innate immune system, with macrophages and neutrophils functioning at 30 hours post fertilization [33]. In contrast, the adaptive immune response only has developed 20 days post fertilization [34]. A recent study showed that zebrafish embryos can be used to investigate the efficacy and mechanism of antifungal drugs. Treatment of the zebrafish embryos with voriconazole showed that this anti-fungal is able to kill hyphae but macrophages are needed to slow down initial fungal growth [35]. We here show no specific differences in these four infection models in virulence between strains that were isolated from invasive aspergillosis infections in humans or from non-invasive sino-nasal infections in dogs [36]. In general, reference strain Af293 was less virulent as compared to the other strains. Analysis of SNPs in virulence-related genes, showed that Af293 differs from the other four strains in 136 SNPs in these genes, one of which in *ftmD* (Afu8g00200) that is part of the fumitremorgin gene cluster. The absence of production of this mycotoxin could explain why Af293 is less virulent as compared to the other strains.

## Results

### Germination dynamics among *A*. *fumigatus* strains

Dormant conidia of *A*. *fumigatus* are surrounded by a protective layer of dihydroxynapthlene (DHN)-melanin and hydrophobin proteins which is shed during germination. As *P*. *aurantium* primarily recognizes swollen conidia of *A*. *fumigatus*, its phagocytic efficiency is highly dependent on the dynamics of the fungal germination process. We therefore first compared the germination kinetics of the five strains by determining the number of germlings at 4, 6 and 8 h. Microscopic analysis of conidia in Czapek-Dox medium (CZD) medium revealed a marked difference in germination kinetics between the five strains (Table 1). Af293 produced relatively few germ tubes (2.6%) after 8 h, when compared to conidia of ATCC46645 of which 54% had formed germ tubes at this timepoint (Table 1). CEA10, DTO271-B5 and DTO303-D3 revealed intermediate dynamics with 39%, 20% and 25% of the conidia forming germ tubes after 8 h, respectively. Apparently, germination kinetics can differ markedly between different fungal isolates and these results clearly demonstrated strongly reduced germination of Af293 in CZD medium.

**Table 1:** Percentage (± SE) of germ tube formation after 4, 6 or 8 h swelling in Czapek-Dox medium (CZD) medium at 37°C.

Strain	4 h incubation		6 h incubation		8 h incubation	
	Conidia	Germlings	Conidia	Germlings	Conidia	Germlings
Af293	100% ± 0	0% ± 0	99.25% ± 0.22	0.75% ± 0.22	97.37% ± 0.26	2.63% ± 0.26
ATCC46645	100% ± 0	0% ± 0	89.69% ± 1.36	10.31% ± 1.36	45.74% ± 2.74	54.26 ± 2.74
CEA10	99.76% ± 0.07	0. 24% ± 0.07	96.7% ± 0.39	3.3% ± 0.39	61.03% ± 1.2	38.97% ± 1.2
DTO271-B5	99.64% ± 0.18	0.36% ± 0.18	97.87% ± 0.33	2.13% ± 0.33	79.98% ± 2.9	20.02% ± 2.9
DTO303-F3	99.25% ± 0.1	0.75% ± 0.1	96.41% ± 0.87	3.59% ± 0.87	75.04% ± 0.74	24.96% ± 0.74

**Fig 1:**
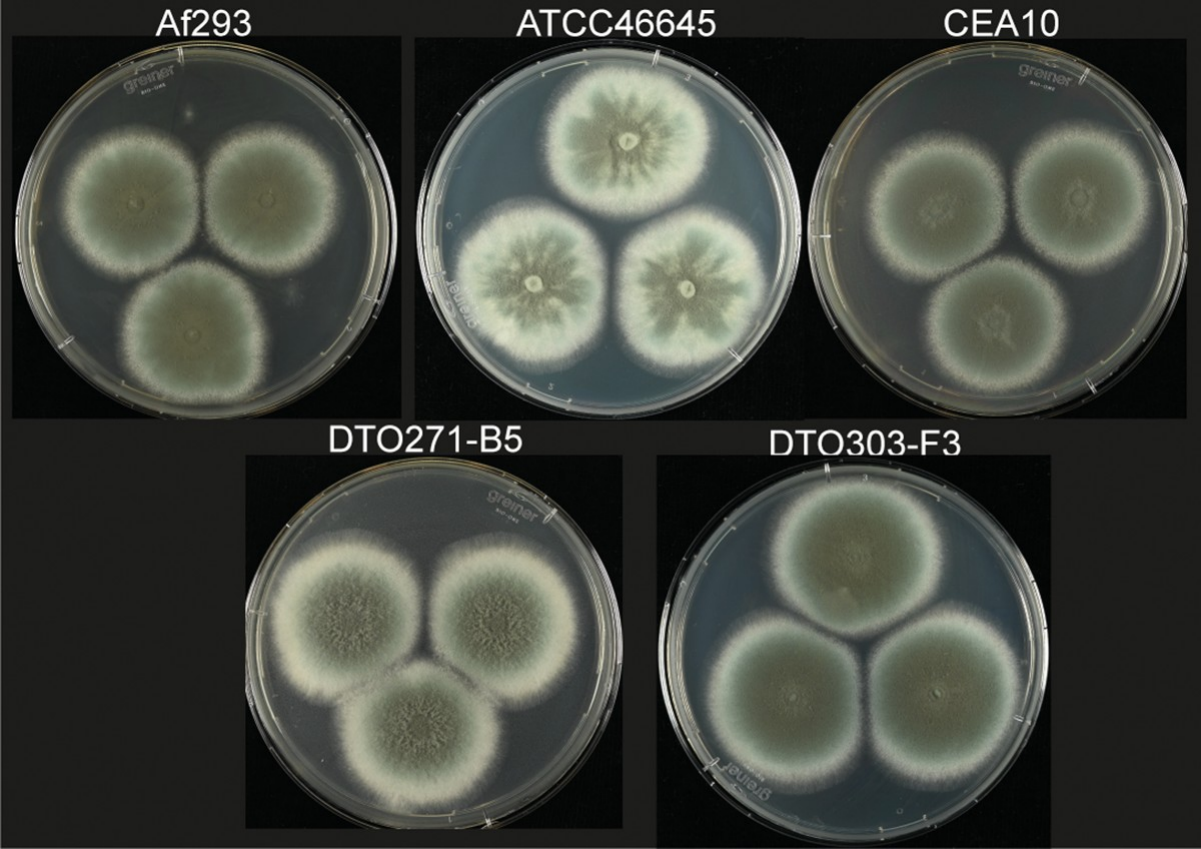
Colony size of *A*. *fumigatus* isolates grown for 2 days on potato dextrose agar (PDA) at 37°C.

Conidia of *A*. *fumigatus* are between 2–3 μm in size [2]. There are also indications that the size of the conidia can differ between strains of the same species [37]. We did not detect significant differences in size of conidia isolated from the five strains used in this study and cultured on PDA (Table 2 and S1 Fig). A three-point inoculation of conidia of the five strains and subsequent growth for 2 days on PDA agar plates did not show major differences in colony size (Table 2). But differences in morphology can be observed (Fig 1). For the colonies of ATCC46645 we see an uneven distribution of the produced conidia, which also seems to be delayed when compared with the other strains. A slight delay in sporulation is also observed with colonies from DTO271-B5. In contrast to the colonies of ATCC46645 we do not see the uneven distribution of conidia, but a light ring at the edge of the colony. Interestingly, Af293, which germinated slower in the CZD medium (Table 1), did not show smaller colonies on PDA after day 1 (not shown) or day 2 (Fig 1).

**Table 2:** Highest frequency of the diameter (μm) of conidia (S1 Fig) and average diameter of colony (mm) (± SE) after 2 days of growth (Fig 1).

Strain	Diameter conidia (μm)	diameter colony (mm)
Af293	2.67	35.08 ± 0.48
ATCC46645	2.67	36.47 ± 0.81
CEA10	2.53	33.39 ± 0.40
DTO271-B5	2.4	37.82 ± 1.08
DTO303-F3	2.53	36.32 ± 1.02

### Variability of sensitivity to oxidative stress between *A*. *fumigatus* strains

**Fig 2:**
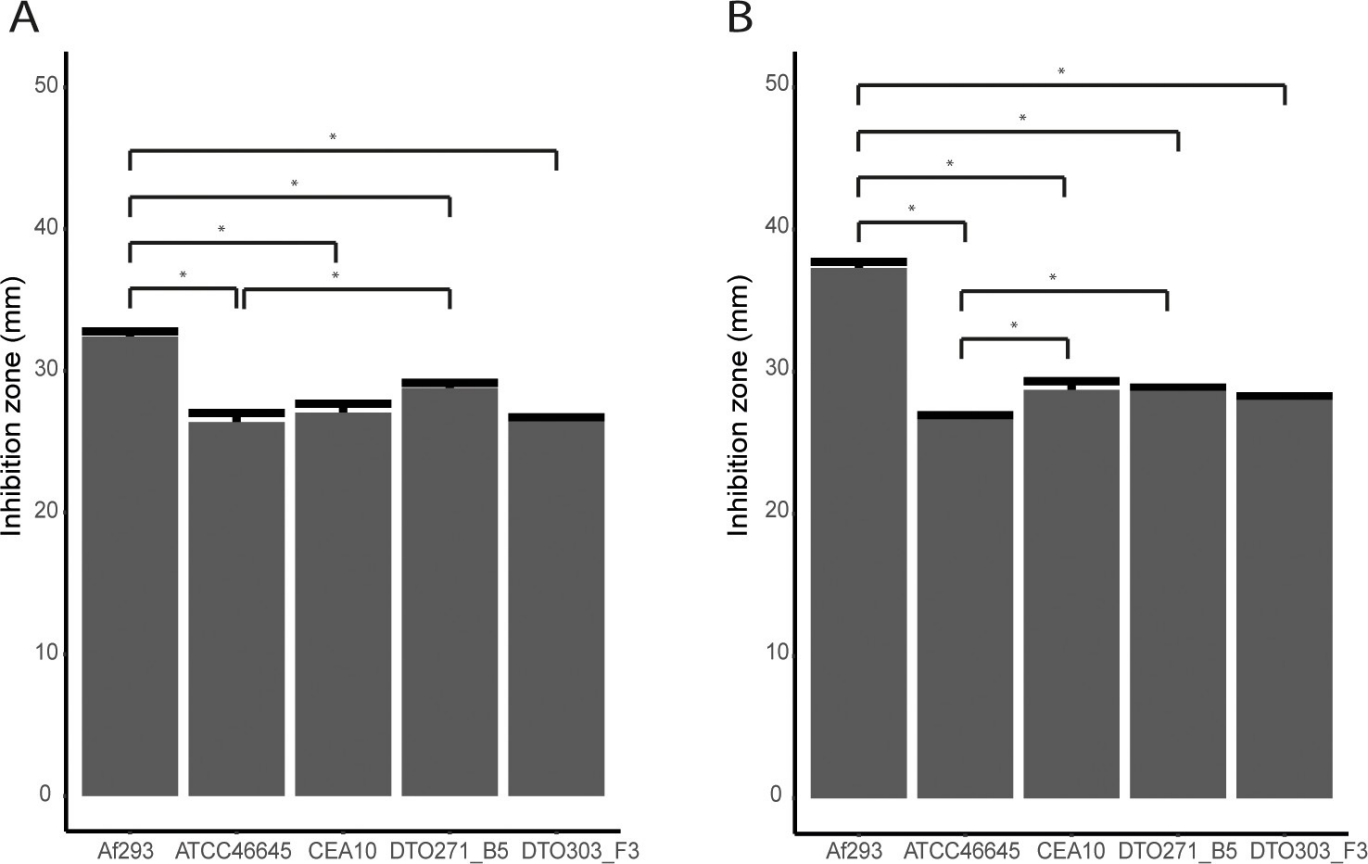
Oxidative stress resistance of *A*. *fumigatus* strains. Sensitivity of five *A*. *fumigatus* isolates for hydrogen peroxide (A, 500 mM H_2_O_2_) (A) or menadione (B, 1 mM Menadione) as determined in an agar plate diffusion assay. Bars represent the average inhibition zone based on biological and technical triplicates (± SE). * indicates statistical significance, based on a one-way ANOVA with a p-value ≤ 0.05.

Strains Af293, ATCC46645, CEA10, DTO271-B5 and DTO303-F3 were compared for their sensitivity to oxidative stress in vitro. To this end, hydrogen peroxide and superoxide (menadione) stress were used since both reactive oxygen species (ROS) are used by immune cells to kill microbial pathogens [38]. Sensitivity to ROS was determined in plate-diffusion assays in which dormant conidia germinate and develop in 16 h. Interestingly, Af293 appeared most sensitive to hydrogen peroxide (Fig 2A) and menadione (Fig 2B), while ATCC46645 and DTO303-F3 were most resistant to both types of stress.

### Interaction of *A*. *fumigatus* strains with lung epithelial cells

**Fig 3:**
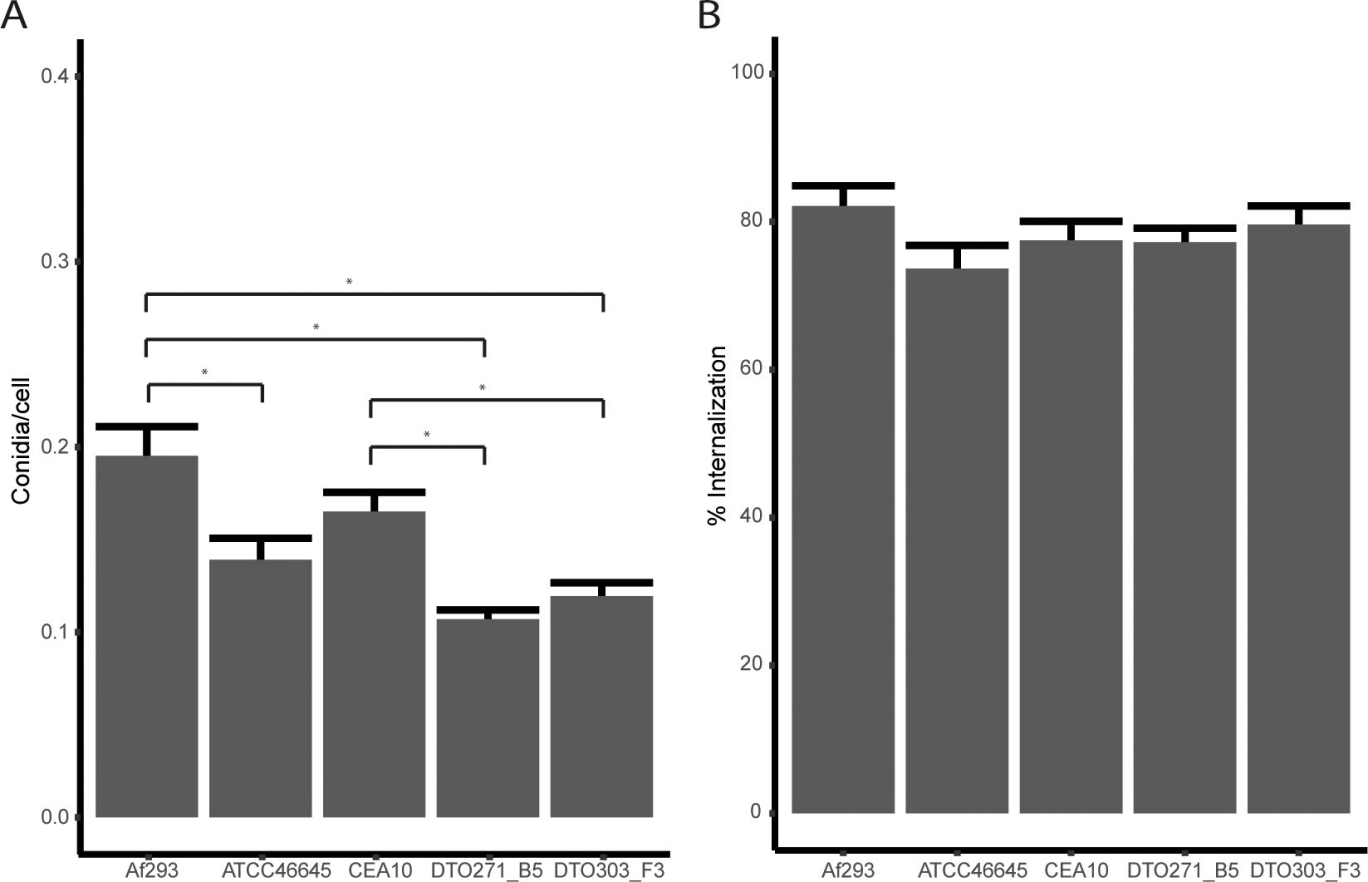
Association (A) and internalization (B) of conidia after 4 h of incubation with type II A549 lung epithelial cells. CalcoFluorWhite (CFW) was used to distinguish between external and internalized fungi. CFW only interacts with chitin in the fungal cell wall if the fungi are is not internalized in A549 cells. Bars represent the average (± SE) of three independent experiments. * indicates statistical significance, for the association data based on a one-way ANOVA, internalization is based upon a Kruskal Wallis test both with a p-value ≤ 0.05.

Conidia were exposed to a monolayer of type II A549 lung epithelial cells. After 4 h of incubation, association (conidia that stay at the epithelial surface after the wash-step) of conidia of Af293 and CEA10 to the A549 lung epithelial cells was highest (Fig 3A; 0.19 and 0.17 conidia/cell, respectively) (representative images can be found in S2A and S2B Fig). The conidia of ATCC46645, DTO271-B5 and DTO303-F3 associated with only 0.14, 0.11 and 0.12 conidia/cell, respectively. Internalization, conidia that associated to the epithelial cells, but were not stained with CalcoFluor White (CFW) of conidia of the five strains hardly varied (representative images can be found in S2C and S2D Fig). The conidia of Af293 and DTO303-F3 were internalized slightly higher (82% and 79%, respectively) compared to CEA10, DTO271-B5 (both 77%) and ATCC46645 (74%), even though these differences were not significant (Fig 3B).

**Fig 4:**
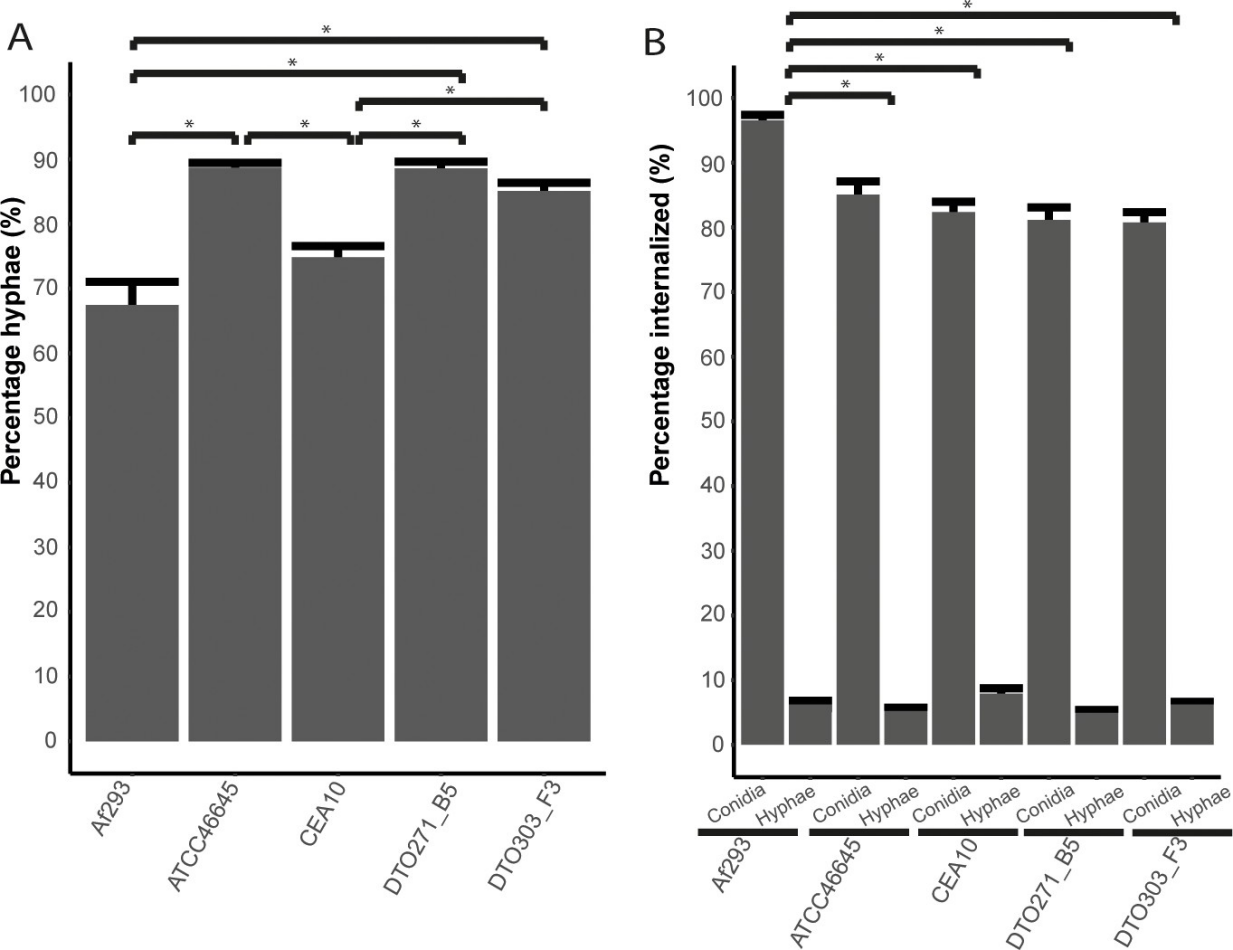
Germination of *A*. *fumigatus* during coincubation with epithelial cells. Percentage of hyphae after 12 h of incubation with type II A549 lung epithelial cells (A). Internalized conidia or hyphae after 12 hours of incubation with the A549 lung epithelial cells (B). Bars represent the average (± SE) of three independent experiments. * indicates statistical significance, based on a Kurskal Wallis test with a p-value ≤ 0.05.

After 12 h of incubation with A549 cells, the majority of the conidia of the five strains had germinated (Fig 4A), germination was determined by the growth of hyphae from the conidia if these hyphae were also stained with CFW they were considered outside (representative images can be found in S3 Fig). The highest germination rate was observed for ATCC46645 (89%), DTO271-B5 (89%) and DTO303-F3 (85%), while lowest germination rate was observed for CEA10 (75%) and Af293 (67%). Most conidia were internalized, whereas most hyphae resulting from these conidia were located outside of the cells and thus had grown outside of the cells (Fig 4B). Of the five strains, conidia of Af293 were most effectively internalized. More germination was observed among conidia outside of the A549 cells, indicating that germination of non-internalized conidia is more efficient then for internalized conidia.

**Fig 5:**
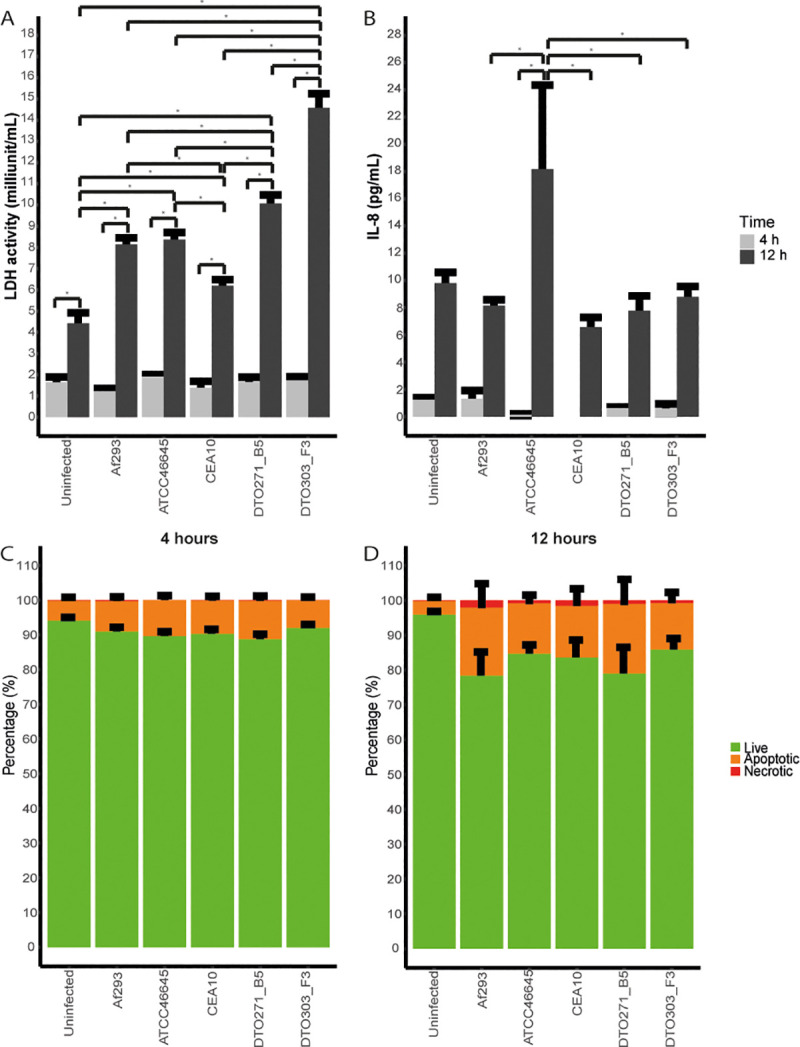
Host cell damage and immune response of A549 cells during confrontation with *A*. *fumigatus*. LDH release (mU mL^-1^) (A) and IL-8 production (pg mL^-1^) (B) of the A549 cells after 4 (light grey) and 12 (dark grey) h of infection. Percentage of cells which are alive (green), apoptotic (orange) or necrotic (red) after 4 (C) or 12 (D) h of infection with *A*. *fumigatus* conidia. Bars represent the average of three independent experiments (± SE). * indicates statistical significance, based on a one-way ANOVA with a p-value ≤ 0.05.

Lactate dehyrogenase (LDH) activity in the medium was determined as measure for host cell damage after 4 and 12 h of co-incubation (Fig 5A). Conidia associated within the first 4 h, and a large part was internalized into A549 cells after 4 h, but no germination was observed. In contrast, after 12 h hyphae are observed, next to conidia, both in and outside the A549 cells. After 4 h, LDH activity in the culture supernatant was similar to the control of uninfected A549 cells for all 5 strains. After 12 h an increase in LDH release into the medium was found when compared to 4 h and to the A549 control at 12 h. A549 cells infected with conidia from DTO303-F3 released most LDH in the medium, indicating that these cells were damaged the most, while A549 cells infected with CEA10 released the least LDH.

The initiation of the immune response in the A549 lung epithelial cells by conidia was determined by measuring interleukin 8 (IL-8) release in the medium. After 4 h of infection hardly any IL-8 release into the medium was found with any of the *A*. *fumigatus* strains (Fig 5B). In contrast, an increase in IL-8 release was found after 12 h, but we did not find a significant difference between the uninfected control and the A549 cells incubated with the *A*. *fumigatus* strains. This shows that, even though most cell damage was induced after infection with DTO303-F3 conidia, this does not correspond to a higher initiation of the immune response measured by IL-8 release.

To determine if the integrity of the epithelial cells was compromised due to interactions with *A*. *fumigatus*, the percentage of apoptotic and necrotic cells was scored after 4 and 12 h using a dual acridine orange and ethidium bromide staining and microscopic analysis (Fig 5C and 5D). Representative image can be found in S4 Fig. Cells were considered healthy when the acridine orange was able to bind to the DNA in the cells, making the cells green. Orange cells were considered in the state of apoptosis, where the acridine orange could also bind RNA. When the ethidium bromide was also able to enter the cells, the cells will colour red, and were scored as necrotic cells. A similar distribution of apoptotic (5–11%) and living cells was observed after 4 h of incubation of A549 cells with or without conidia, whereas virtually no necrotic cells were detected. In contrast, a clear increase in apoptotic cells was observed after 12 h co-incubation with conidia (Fig 5D). Conidia of Af293 and DTO271-B5 might induce a slightly higher apoptosis (20% and 19%, respectively) and necrotic (2% and 1%, respectively) incidence of A549 cells as compared to the other three strains (around 15% apoptotic and 0.3% necrotic) but these differences were not significant.

Together, it is concluded that a 12 h incubation of A549 with *A*. *fumigatus* conidia results in an increase in apoptotic cells, but the 5 different strains did not induce significant differences in the amount of apoptotic and necrotic cells. Moreover, no major differences in internalization, germination, cell damage or IL-8 release were found between strains isolated from an invasive infection (Af293, ATCC46645 and CEA10) and strains derived from a non-invasive infection (DTO271-B5 and DTO3030-F3). The latter two strains did however associate to a lower extend to A549 cells. We did see that conidia of Af293 associated and internalized more to A549 cells but germinated less. However, induced cell damage did not significantly differ between the strains.

### Internalization and killing of *A*. *fumigatus* strains by *Protostelium aurantium*

**Fig 6:**
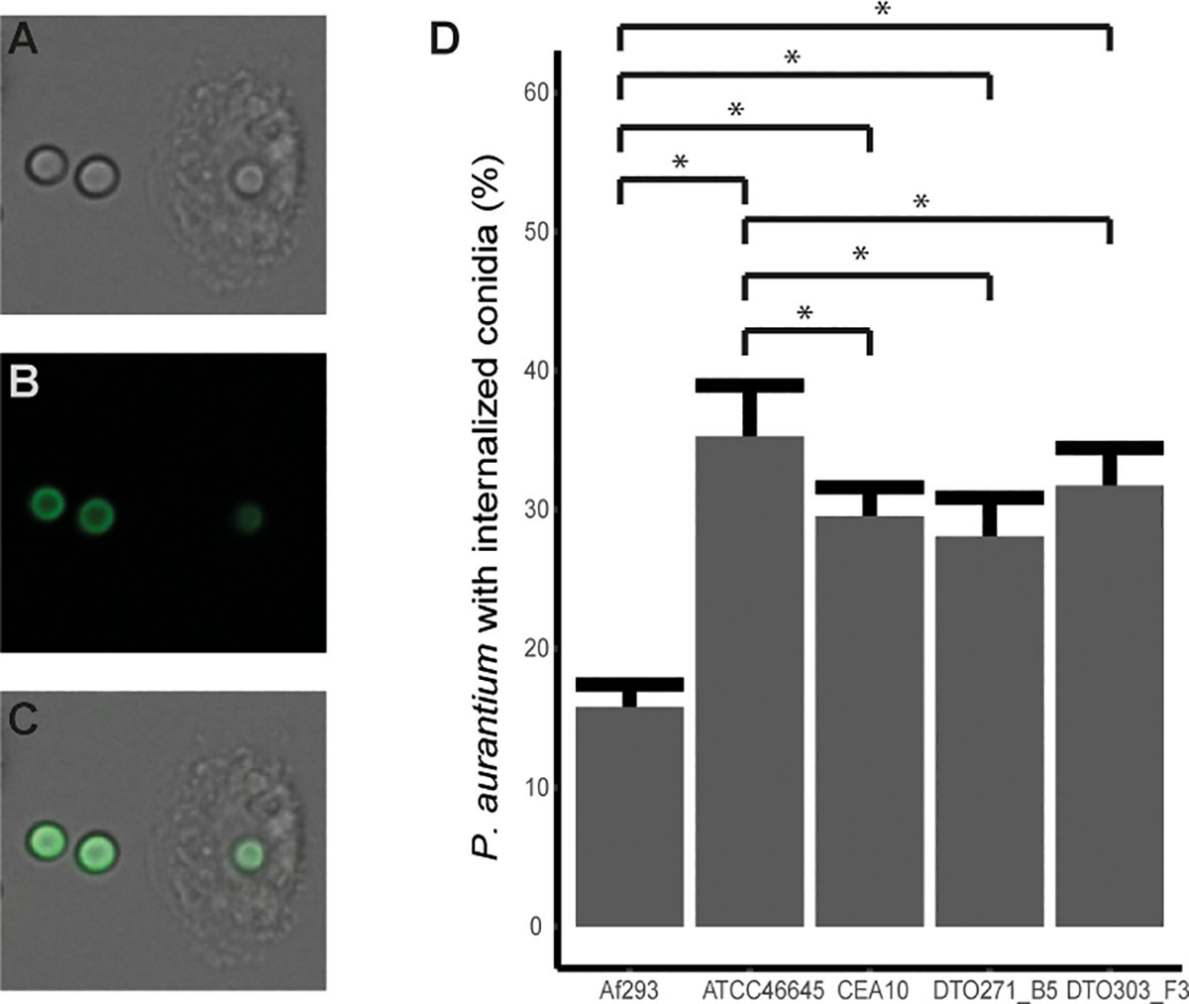
Uptake of swollen conidia by *P*. *aurantium* after 2 h of incubation. DIC (A) and FITC (B) channel of phagocytosed and non-phagocytosed pre-incubated conidia (4.5 h) and their merge (C). FITC signal of the phagocytosed conidia is lower due to the acidification of the phagolysosome. D) Percentage (± SE) of amoeba that had phagocytosed one or more conidia. Bars represent three individual experiments. * indicates statistical significance, based on a Kruskal Wallis test with a p-value ≤ 0.05.

Phagocytic uptake and survival of the fungus was monitored when exposed to the amoebae at different time points of its germination process. Internalization of conidia can be monitored due to quenching of FITC fluorescence in the acidic compartment of the phagolysosome of the amoebae (Fig 6A–6C) [39]. At 4.5 h after inoculation of conidia into CZD medium to start germination, conidia of ATCC46645 were taken up with the highest efficiency, whereas uptake of Af293 conidia had the lowest efficiency (Fig 6D). The fact that Af293 uptake was approximately 50% lower as compared to the other four strains reflected its less effective recognition by *P*. *aurantium*, which could due to a delayed germination.

**Fig 7:**
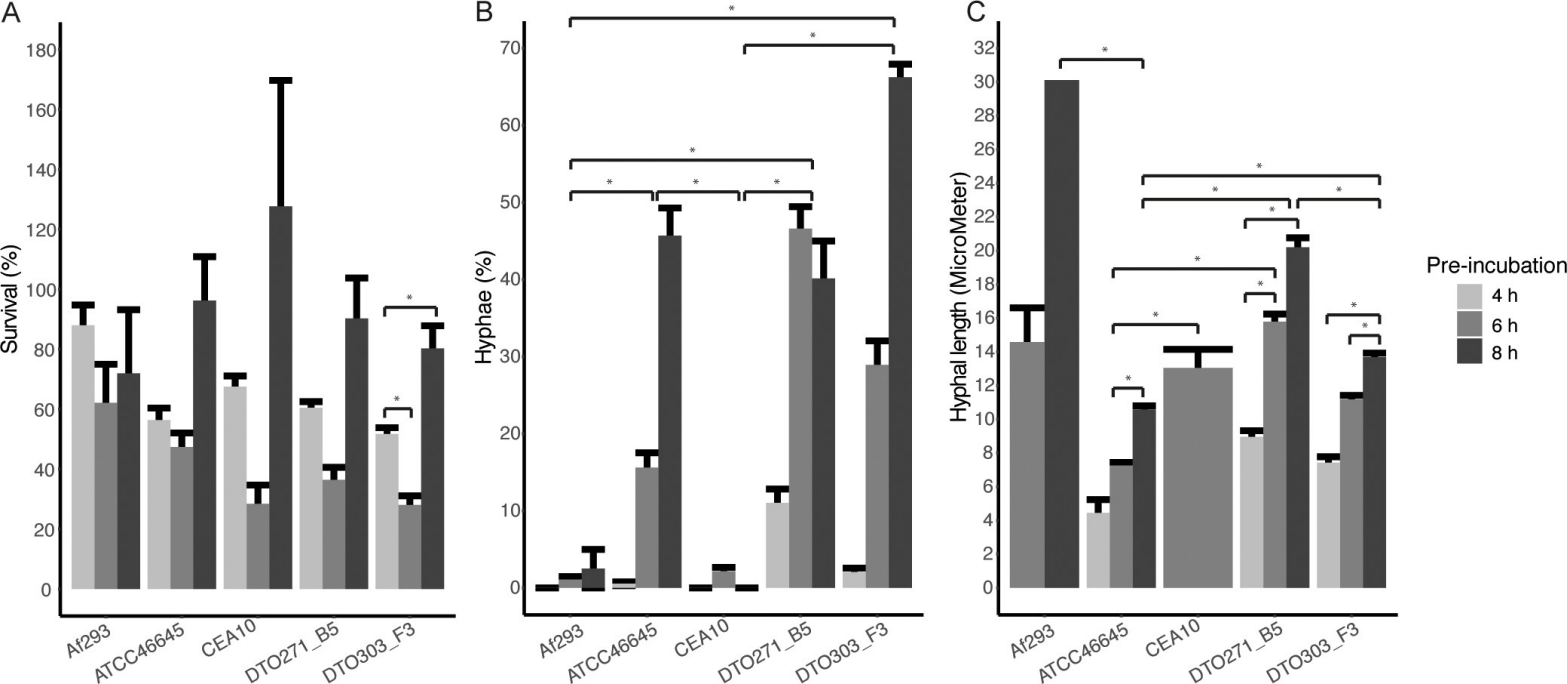
Resistance of *A*. *fumigatus* to the predatory phagocyte *P*. *aurantium*. Survival, measured by the conversion of resazurin to resorufin (A), hyphal growth (B) and hyphal length (μm; C) of conidia following pre-incubation for 4 (light grey), 6 (grey) or 8 h (dark grey) and coincubation with *P*. *aurantium* for 18 h. Bars represent the average (± SE) of three independent experiments. * indicates statistical significance, survival and hyphal growth is based on a Kruskal Wallis test and the hyphal length is based on a one-way ANOVA, both with a p-value ≤ 0.05.

Next, survival of 4, 6 and 8 h pre-incubated conidia was quantified after exposure to amoebae. Therefore the amount of resazurin converted to resorufin by the fungi was measured. For all five strains, the early time-point of 4 h resulted in higher survival rates when compared to conidia that had been pre-incubated for 6 h, and thus underwent an extended swelling period (Fig 7A). However, this effect was not significant for the ATCC46645 strain. Highest survival rates were seen when the fungal conidia were pre-incubated for 8 h (Fig 7A). This is in line with the high number of germ tubes formed that will interfere with the phagocytic uptake an attenuate phagocytic killing (Table 1). Conidia of Af293 behaved different when compared to the other 4 strains. Survival of 4 h pre-incubated conidia was higher than for the other 4 strains, while 8 h pre-swollen Af293 conidia were killed more efficiently than any of the other four strains.

We further monitored germination and growth of 4, 6 and 8 h pre-incubated conidia after an 18 h incubation period with the amoeba. The number of conidia and hyphae were scored microscopically by the number and length of the hyphae. While for ATCC46645, DTO271-B5 and DTO303-F3 the number of hyphae increased with a prolonged period of pre-incubation, only few hyphae were found for Af293 and CEA10, regardless of how long these conidia had been pre-incubated (Fig 7B). For Af293, those hyphae that were found continuously grew in the presence of the amoebae (Fig 7C). For CEA10, conidia hardly germinated after an 18 h interaction with the amoebae (Fig 7B) and thus, essentially no hyphae were detected for this strain to monitor hyphal length (Fig 7C). Germination of DTO271-B5 and DTO303-F3 was also reduced in the presence of the predator, but only a slight retardation in germination was observed for ATCC46645. These results indicate that the amoebae can affect the germination process and the formation of germ tubes via an unknown mechanism. For Af293 the germination process was found to be relatively slow which was further aggravated in the presence of *P*. *aurantium*.

**Fig 8:**
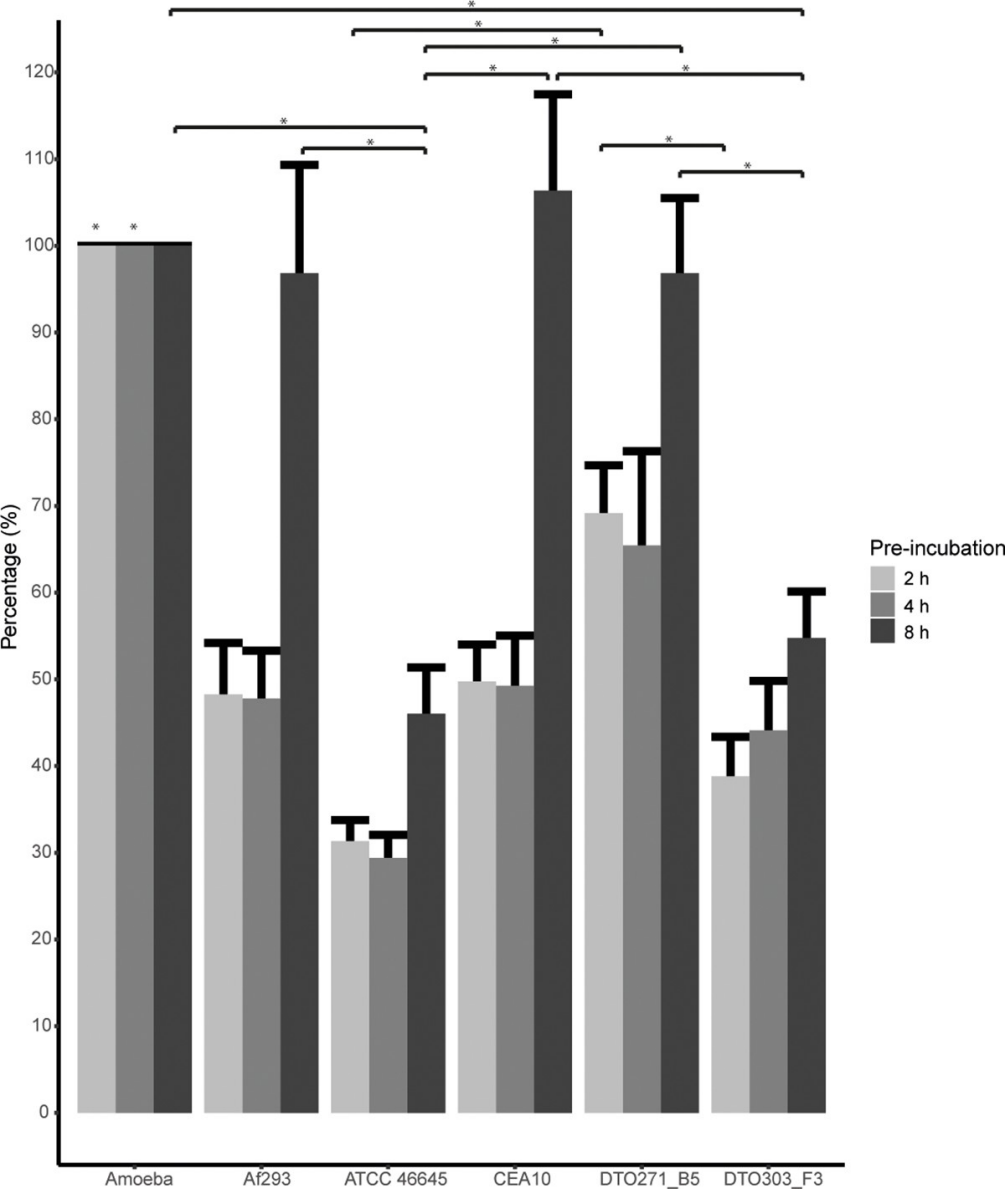
**Amoeba survival after 18 hours of co-incubation with conidia following pre-incubation for 4 (light grey), 6 (grey) or 8 (dark grey) hour swollen conidia.** Bars represent 3 individual experiments ± SE. * indicates a statistical difference, based upon the Kruskal Wallis test.

Next survival of the amoebae was monitored by counting the amoebae before and after the 18 h incubation with conidia at different stages of germination. Survival of amoeba was virtually 100% when exposed to 8 h pre-incubated conidia of Af293, CEA10 and DTO271-B5, whereas approximately 50% of the amoeba survived incubations with 8 h pre-incubated conidia of ATCC46645 and DTO303-F3 (Fig 8). These results indicate that ATCC46645 and DTO303-F3 had the highest amoebicidal activities. Remarkably, survival of the amoebae did not correlate with germination and hyphal length. Amoebae incubated with conidia of Af293, CEA10 and DTO271-B5 survived the best, while only the latter strain germinated efficiently (Fig 8). Together, these results indicate a marked difference between the behaviour of Af293 in the amoeba infection system in comparison with the other four isolates differing in efficiency of uptake, killing by amoebae and germination. The apparent low level of initiation of germination of Af293 most likely results in relative low uptake and high survival. In contrast, higher germination kinetics of the other four strains results in more efficient uptake and killing. Together, these results indicate that delayed germination, as demonstrated for Af293, may protect conidia during early confrontation with a phagocyte.

### Galleria model

**Fig 9:**
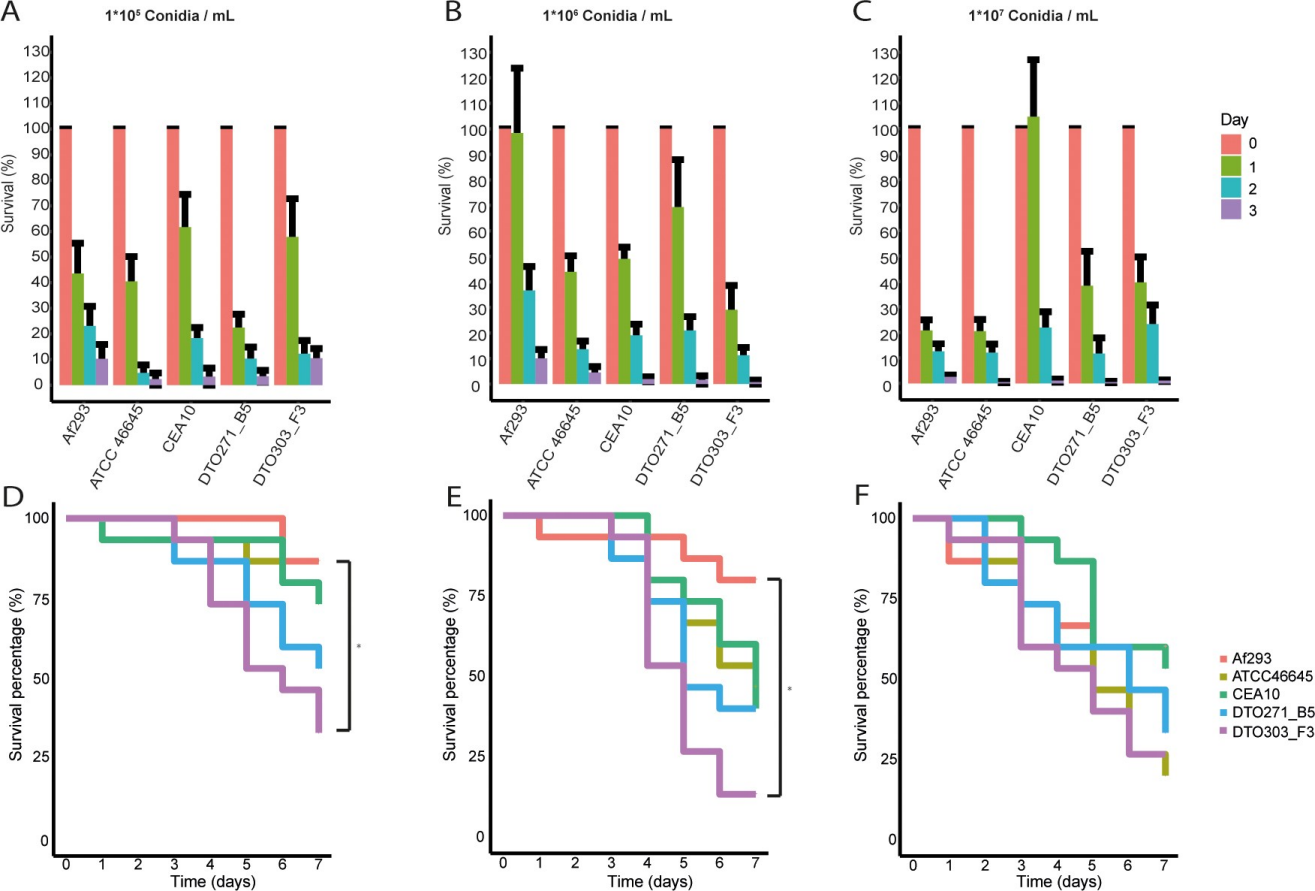
Survival of *A*. *fumigatus* (A-C) and *G*. *melonella* larvae (D-F) after infection. Bar graph of *A*. *fumigatus* survival and Kaplan-Meier survival plots of *G*. *melonella* over 3 or 7 days of incubation with 10^5^ (A, D), 10^6^ (B, E) and 10^7^ (C, F) conidia mL^-1^, respectively. Bars represent the average (± SE) of three independent experiments. Each line consists of three biological replicates. * indicates statistical significance, fungal survival is based on a Kruskal Wallis test and the larvae survival on a non-parametric log rank test, both with a p-value ≤ 0.05.

Virulence of the five *A*. *fumigatus* strains was assessed in *G*. *melonella*. To this end, larvae were injected in the hindleg with 10^5^, 10^6^ and 10^7^ conidia mL^-1^ and incubated at 37°C. We first followed the survival of the *A*. *fumigatus* strains in the *G*. *melonella* larvae over the first 3 days of infection (Fig 9A–9C). We found a general decrease in the number of conidia in the haemolymph of the larvae, which was irrespective of the strain or spore concentration used, indicating that conidia exit the haemolymph quickly, and with the similar efficiencies to establish the infection elsewhere in the larvae. The slower germinating Af293 was found to be retained in the haemolymph at day 1, but only when larvae were challenged with an intermediate number of conidia. Also, CEA10 was retained for 24 h upon infection with highest conidial load.

Next, larvae survival was monitored for 7 days after the injection. At the highest dose of 10^7^ conidia mL^-1^, all strains were able to develop infections in the *Galleria* larvae killing 50–75% of the larvae (Fig 9F). Larvae survived better when exposed to 10^6^ conidia mL^-1^ of Af293, CEA10 and ATCC46645 (Fig 9E) and killing remained high for DTO271-B5 and DTO303-F3, even at the lowest infection dose of 10^5^ conidia mL^-1^ (Fig 9C). Taking the results of the three spore amounts together, larvae injected with conidia of slower germinating Af293 survived the best when compared to the other four strains. However, virulence with Galleria was not entirely dependent on germination kinetics, as also the faster germinating strain CEA10 showed reduced killing. Together, these results show that larvae were killed irrespective of the initial fungal load and killing probably followed germination of fungal conidia outside the haemolymph system. Overall killing of the larvae was most efficient for ATCC46645, DTO271-B5 and DTO303-F3 indicating more specific virulence determinants for these three strains.

### Zebrafish model

**Fig 10:**
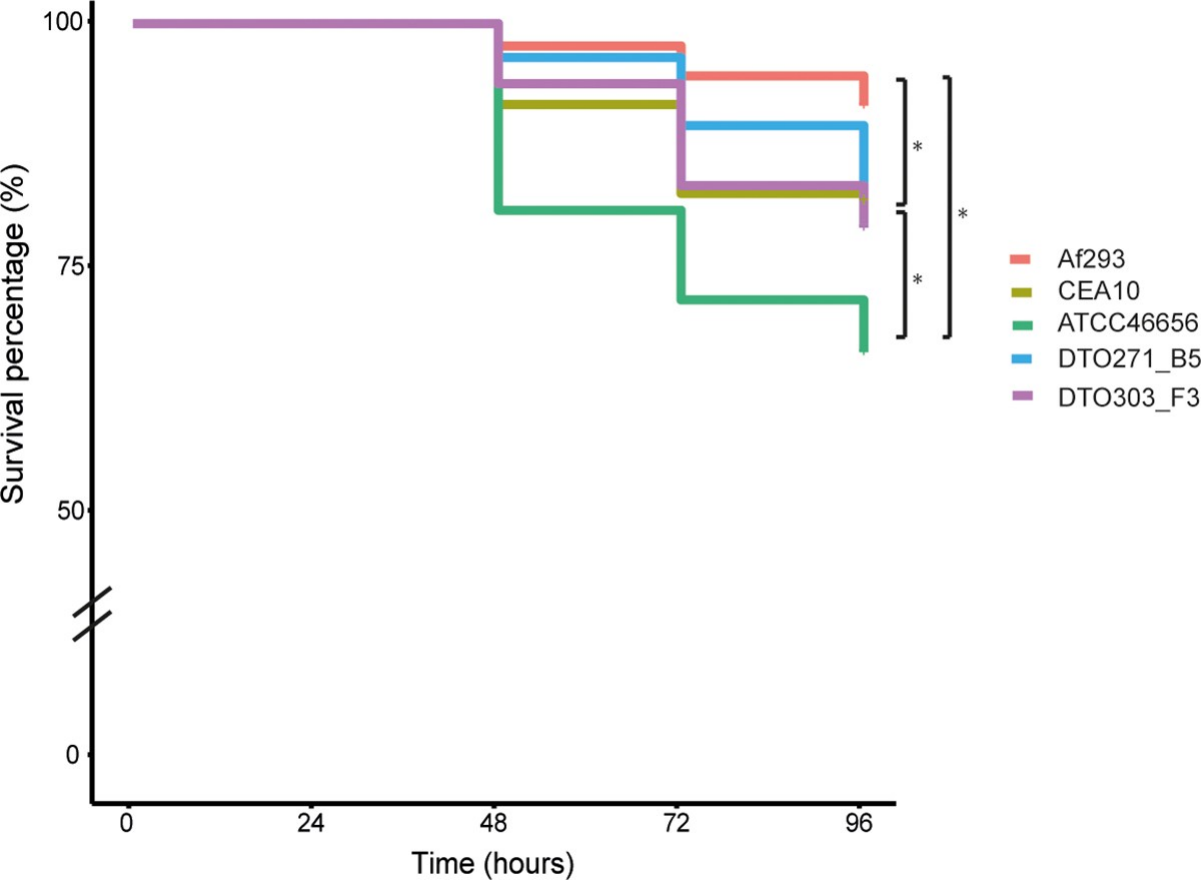
Kaplan-Meier survival plot of the zebrafish embryos injected with 100 conidia of different *A*. *fumigatus* isolates. Each line consists of three biological replicates. * indicates a statistical difference, based on a non-parametric log rank test with a p-value ≤ 0.05.

Zebrafish embryos were the fourth model used to assess virulence between the five *A*. *fumigatus* isolates. Embryos were injected at 36 h post fertilization. At this time-point primitive macrophages are present and after the start of infection primitive neutrophils start to differentiate. This means that the embryos rely on their innate immune system to clear the infection [40]. Injection of 100 conidia into the hindbrain of the zebrafish embryos led to mortality of the embryos over a 96 h period. The mortality of the zebrafish embryos was determined if we could still see a heartbeat in the embryo. The highest and lowest survival was observed with Af293 and ATCC466645 conidia, respectively (Fig 10).

### Virulence-related genes

**Fig 11:**
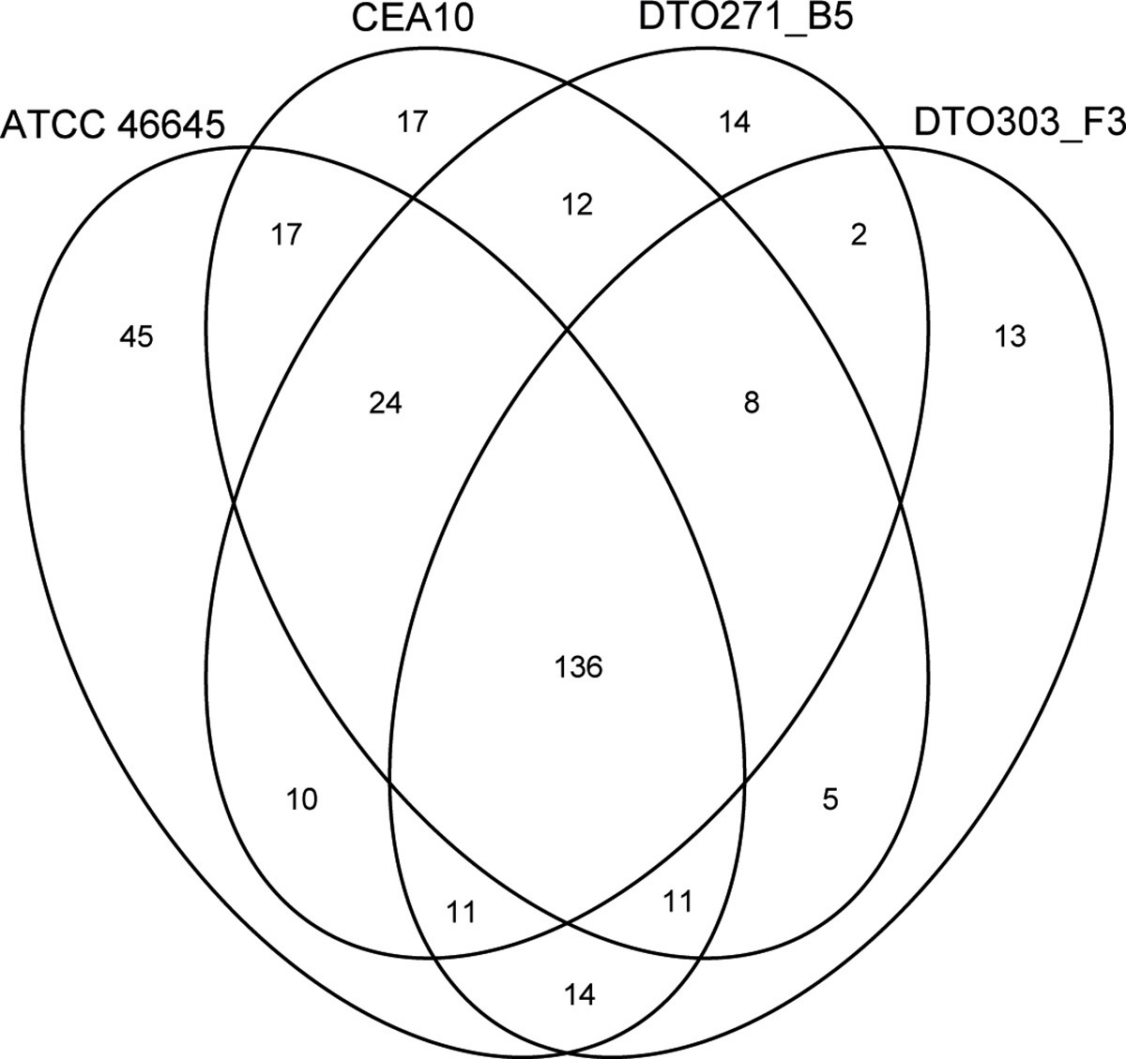
Venn diagram of all SNPs (low, moderate and high impact) in the virulence related genes listed by [41]. The genome of Af293 is used as a reference genome.

Results demonstrate that Af293 is less virulent in amoebae, *Galleria* and zebrafish infection models as compared to the other four strains. ATCC46445 and DTO303-F3 were most virulent in zebrafish and *Galleria* infection models, respectively. This variation in virulence might be related to absence or presence of virulence-related genes or mutations in these genes. Single Nucleotide Polymorphisms (SNPs) were identified within the set of previously published virulence-related genes [41] (Fig 11). The virulence-related genes are divided in sub-groups of genes related with thermotolerance, resistance to the immune response, cell wall, toxins and secondary metabolites, allergens, nutrient uptake and signalling and regulation (S1 Table). Surprisingly, there were no high impact SNPs (defined as SNPs predicted to have a high impact on the protein such as loss of function, as predicted by Snpeff [42] present in the selected genes. Strikingly, 136 SNPs were identified that were shared between the four strains, which indicates that Af293 differs with the other strains in 105 virulence-related genes. In addition, ATCC46645 stood out with 45 unique SNPs as compared to the other three strains, when aligned to the Af293 reference genome. CEA10, DTO271-B5 and DTO303-F3 had 17, 14, and 13 unique SNPs, respectively, when aligned to the Af293 reference genome. Strain specific mutations in virulence-related genes might contribute to difference in virulence between these strains. The 136 SNPs shared between ATCC46645, CEA10, DTO271-B5 and DTO303-F3 occurred in all groups of virulence-related genes. For instance, SNPs were identified in the fumagillin biosynthesis polyketide synthase fma-PKS (Afu8g00370), (S2 Table). *A*. *fumigatus* strains that do not produce fumagillin, due to a *fmaA* knock-out, cause less damage to pulmonary epithelial cells when compared to the wild-type strain [43]. The observed SNPs could alter the fumagillin production in ATCC46645, CEA10, DTO271-B5 and DTO303-F3 or especially in Af293 and affect virulence of the latter strain, but more analysis is required to confirm this.

Conidia of ATCC46645 and DTO303-F3 were the most virulent based on survival data of *G*. *melonella* larvae and zebrafish embryos. These strains share 14 SNPs (Fig 10; S3 Table) in genes belonging to resistance to the immune response, cell wall, toxins and secondary metabolites and allergens. For instance, there were SNPs in the superoxide dismutase gene *sod4* (Afu6g07210), the class I chitin synthase *chsA* (Afu2g01870), the Asp-hemolysin gene *aspHS* (Afu3g00590) and in a gene with predicted nucleic acid and zinc binding activities (Afu5g12760). Most SNPs were found in genes belonging to the fumigaclavine C (fga) biosynthesis cluster. In this gene cluster we see SNPs in *fgaFS* (Afu2g17970), *easM* (Afu2g18010), *fgaAT* (Afu2g18020) and *fgaCAT* (Afu2g18030). Fumigaclavine C has been reported to have anti-inflammatory effects [44] and reduces the expression of inflammatory cytokines [45]. The two observed shared SNPs, which lead to an amino acid change in the genes related to the fumigaclavine C biosynthesis cluster could affect the anti-inflammatory effects of the fumigaclavine C. This could lead to an increase in the inflammatory response and cytokine production of the host and therefore leading to a more severe infection and killing of the host.

**Fig 12:**
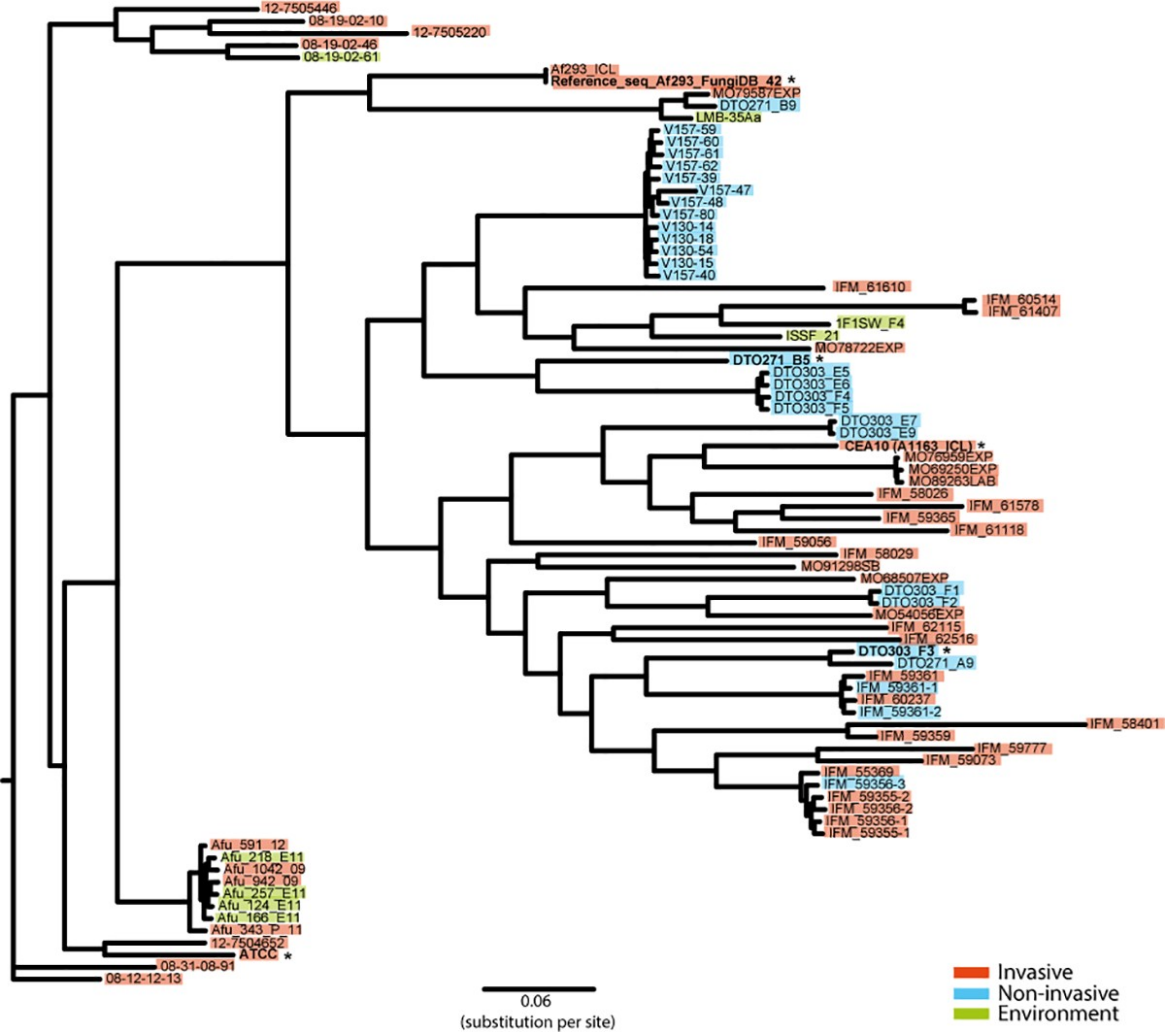
Phylogenetic tree based on SNPs compared to the Af293 reference genome. Strains that were isolated from an invasive infection are marked red, from a non-invasive infection marked blue and strains isolated from the environment marked green. Strains used for the experiments in this study are indicated in bold with an *.

A phylogenetic tree based on all the SNPs in the genome of the strains listed in S4 Table was made based on the Af293 reference genome (Fig 12). The *A*. *fumigatus* strains can be found in three clusters. Where Af293, CEA10, DTO271-B5 and DTO303-F3 are located in the same cluster, in this cluster CEA10, DTO271-B5 and DTO303-F3 are closest to each other. ATCC46645 is located in a separate cluster. The invasive and non-invasive isolates, as well as environmental isolates, do not cluster together. But we do see a cluster of non-invasive strains, that originate from samples taken at different time points from the same patient with a chronic pulmonary aspergillosis [46]. Strains isolated from dogs with a non-invasive sino-nasal aspergillosis [47] generally also cluster when they had been isolated from the same dog.

## Discussion

**Fig 13:**
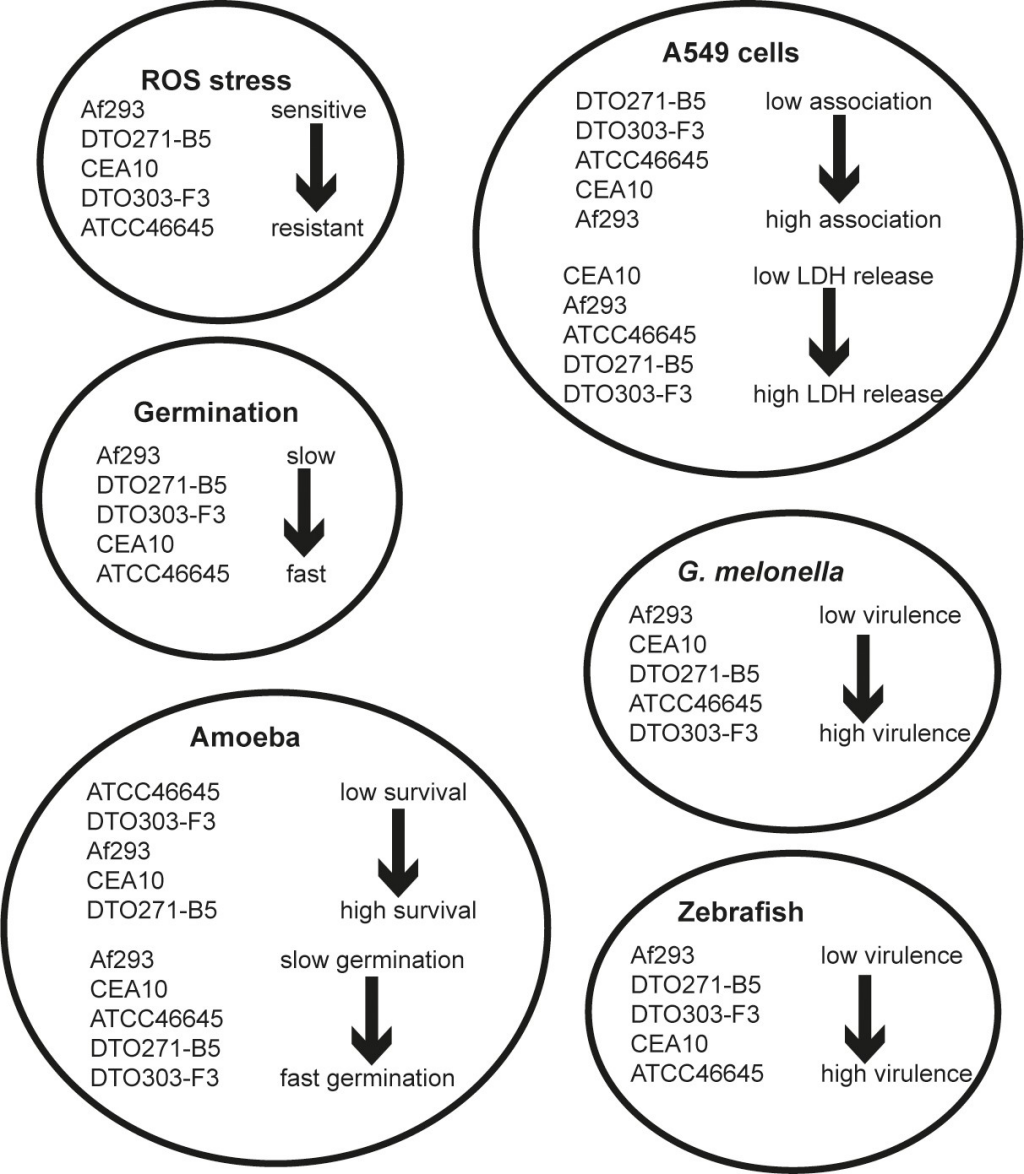
Summary of the main findings. A complete overview can be found in S5 Table.

In this study we have addressed the predictive value of different fungal infection model systems, to see if these models can predict the virulence of *A*. *fumigatus* isolates. Previous studies have indicated that genetic and phenotypic variability between *A*. *fumigatus* isolates impact virulence [16,17,19]. Here, we compared five isolates of *A*. *fumigatus* that were isolated from patients with invasive (Af293, CEA10 and ATCC46645) or non-invasive aspergillosis (DTO271-B5 and DTO303-F3) in four infection models. An overview of the results is presented in Fig 13 and S5 Table. The most striking result is the observation that virulence of individual *A*. *fumigatus* strains varies significantly between the four infection models. We observed that Af293 is less virulent as compared to the other four strains. No differences were seen between virulence of the strains in type II A549 lung epithelial cells. In contrast, strain ATCC46645 was most virulent in the amoeba and zebrafish model, whereas DTO303-F3 was the most virulent in the *Galleria* infection model.

In plate diffusion assays with either H_2_O_2_ or menadione Af293 appeared most sensitive to these oxidative stress conditions, whereas ATCC46645 is the most resistance. In this plate assay the stressor diffuses into the plate and creates an increasing gradient of ROS which is exposed to dormant and germinating conidia in time. The difference in sensitivity of different strains is a reflection of their individual resistance mechanisms. Furthermore, differences in sensitivity between the strains can also be due to their differences in kinetics and low extent of germination. Germlings of slow germinating strains might actually be exposed to an increased concentration of ROS formed in time by diffusion. We have not compared the germination kinetics in minimal medium that were used in these experiments but cannot exclude the possibility that slow kinetics of germination is actually responsible for the increased ROS sensitivity of Af293 rather than a difference in resistance mechanism (e.g. catalases). In this context it is important to emphasize that Af293 showed strongly reduced germination in CZD medium as compared to the other four strain (Table 1).

The amoeba infection system is regarded as a model system for phagocytosis by macrophages. Reduced uptake and high survival can be regarded as evasion of phagocytosis. Interestingly, 4 and 6 h pre-swollen conidia of Af293 stand out as compared to the four other strains, surviving the best and having the lowest uptake. This would suggest that Af293 is actually better in evading the immune system as compared to the other four strains. The interaction studies with A549 lung epithelial cells indicate no major differences between the five strains, except for a slightly higher efficiency of association of Af293 conidia. Together with an internalization of 80% this results in a slightly higher uptake of conidia in the A549 lung epithelial cells. Internalized conidia of *A*. *fumigatus* were not killed by amoebae and germinated much less in amoeba when compared to A549 lung epithelial cells. These internalized conidia therefore represent a potential reservoir of these fungal strains. In addition, delayed germination after uptake in epithelial cells, observed by Escobar et al., 2016 [20], can also be regarded as a strategy to escape immune surveillance in lungs, for example by alveolar macrophages at the lung surface.

Af293 behaves rather different in the four infection models when compared to the other strains. It has reduced virulence and escapes immune recognition. This is in accordance with previous work showing that Af293 was less virulent as compared to CEA10 in an IPA murine infection model and in zebrafish [18,19]. In this context it is interesting to notice that Af293 is clearly more sensitive to in vitro ROS stress conditions. Evading phagocytosis is a way to escape killing by ROS, so Af293 benefits especially by escaping uptake by phagocytic cells. This trait seems to be correlated with the efficiency of germination. Dormant conidia of Af293 hardly germinate in CZD medium, while conidia of Af293 and CEA10 hardly germinate in presence of amoebae. The absence of germination of CEA10 and Af293 and the reduced germination of DTO303-F3, DTO271-B5 and ATCC46645 in the presence of *P*. *aurantium* and type II A549 lung epithelial cells therefore appears to be an inhibitory mechanism induced by the host. Af293 stands out in this comparison as germination is very low and possibly the increased sensitivity of Af293 to hypoxic conditions is involved [18]. Reduced availability of oxygen in the systems affects the radial growth on plate and the biomass production in a liquid culture and could also influence the ability to initiate germination. Germination in hypoxic condition could be influenced by changes in gene expression or metabolism [48]. Differences in colony morphology are also observed for colonies from ATCC46645 (Fig 1), but we also see that conidia of this strain are virulent in the *G*. *melonella* and zebrafish infection model. This could indicate that ATCC46645 is better adapted to hypoxic conditions and that this explains the altered morphology and the increase in virulence.

Absence of germination also results in reduced recognition by immune cells since antigenic surface components are more shielded by cell wall surface components in resting conidia. The presence of DHN-melanin, rodlets and the stealth protein CcpA are, in part, responsible for shielding conidial surfaces [49]. Absence of CcpA was previously shown to reduce virulence in a neutropenic mouse infection model. This was correlated with enhanced recognition of 3 h swollen conidia of the *ccpA* deletion strain by primary human polymorphonuclear neutrophils (PMNs) and activation of human monocyte derived dendritic cells. In addition, absence of CcpA resulted in reduced cell damage of A549 lung epithelial cells, while uptake of swollen conidia by A549 lung epithelial cells was similar. We studied binding and uptake of dormant conidia of Af293 by A549 lung epithelial cells and observed a higher association as compared to the other four strains while only a slightly higher cell damage was induced after 12 h by Af293. Whether recognition of the dormant conidia by A549 lung epithelial cells is modulated by CcpA requires further study. Other research indicates that exposed β-glucan and DHN-melanin in dormant conidia are already sufficient for recognition [50–53]. On the other hand, recognition and uptake of conidia by amoeba does require pre-swelling of the conidia. We observed that uptake of pre-swollen Af293 conidia is 50% reduced as compared to the other four strains. This observation suggests that Af293 has a modified conidial surface, for example due to differences in the amount of CcpA or that swelling of Af293 conidia is less efficient. The latter supposition is in agreement with the observed reduction in formation of germlings after 8 h incubation in CZD medium. Even though these characteristics of Af293 seem to be beneficial for its virulence, it was found the be the least virulent in *G*. *melonella* and the zebrafish infection model. This indicates virulence of the fungus in these models is multifaceted with host interactions at several levels of complexity, such as germination dynamics, recognition by host cells, as well as the site of primary infection in the host.

Trypacidin is produced by the polyketide synthase TynC and present on the conidia of *A*. *fumigatus*, except on CEA10 conidia, which due to a frameshift in the TynC gene cluster does not produce trypacidin [54]. Deletion of *tynC* abolishes production of the toxin trypacidin, increases phagocytosis by macrophages and the amoeba *Dictiostelium discodeum*, and increases survival of the amoeba after infection [55]. In our *P*. *aurantium* amoeba model we observed an increase in survival of the tested strains of 4 h pre-swollen conidia compared to 6 h pre-swollen conidia (Fig 7A). Trypacidin might be lost from the conidia upon swelling and this could decrease the uptake by amoeba and, in addition, a decrease in killing. Interestingly, 8 h pre-swollen conidia of the ATCC46645 and DTO303-F3 strains are actually more effective in killing amoeba. This suggests that trypacidin is most likely not involved but other killing mechanisms are functional in these two strains at this time-point, for example via different mycotoxins like gliotoxin or fumagilin [43,56]. *A*. *fumigatus* strains lacking fumagillin cause less damage to epithelial cells when compared to the wild-type strain [43]. It should be mentioned that production of trypacidin is temperature dependent and is high when the fungus is grown at 25°C but very low at 37°C [57], the latter being the temperature used in this study to culture the fungal strains. We expect that the amount of trypacidin is low but might vary between the five strains. Further research is required to assess this difference.

Gliotoxin has been shown to be an important determinant for virulence in the *G*. *melonella* infection model [29]. An increase in gliotoxin production by *A*. *fumigatus* led to a decrease in survival of *G*. *melonella* larvae. We showed that the virulence of Af293 conidia is lower in *G*. *melonella* larvae and zebrafish embryos, but also that this strain has less germlings after an 8 h incubation period in CZD medium. The amount of LDH released in the medium by A549 cells is also low after 12 h of co-incubation, which indicates that the slower germination of Af293 could be the reason for the decrease in virulence. Future research should determine gliotoxin production and virulence of the strains used in this study correlate [29]. Another mycotoxin produced by *Aspergillus* species is fumitremorgin. Af293 contains a mutation in *ftmD* (Afu8g00200), which is part of the fumitremorgin gene cluster and essential for production of fumitremorgin C [58]. A point mutation replacing arginine by a leucine at position 202 of the protein is responsible for the lack of fumitremorgin C production (Kato et al., 2013, S6 Table). It is possible that the absence of this mycotoxin in Af293 contributes to the decreased virulence compared to ATCC46645, CEA10, DTO271-B5 and DTO303-F3, but further research is required to verify this.

Previous research showed that Af293 is more sensitive to pH stress when compared to DTO271-B5 and DTO303-F3 when grown in medium at pH 8, but it is more resistant to copper stress as compared to these strains [36]. Notably, strains ATCC46645, CEA10, DTO271-B5 and DTO303-F3 have SNPs in the multicopper oxidase *abr1*, when compared to Af293 (S1 Table). Gene *abr1* is described to have a role in the DHN-melanin synthesis gene cluster [59] but its role in copper stress is still unknown. With the SNPs observed in *abr1* and the observed copper resistance in Af293, this could indicate that *abr1* is important for the fungus to cope with copper stress.

In the phylogenetic tree, based on SNPs within the genome, we see that there are no clusters of isolates originating from the environment, invasive or non-invasive infections (Fig 12). It is also observed that Af293, CEA10, DTO271-B5 and DTO303-F3 are located in the same cluster, whereas ATCC46645 is located in a separate cluster. Based on the SNPs in the virulence-related genes we see that the most difference is between Af293 and the other four strains (Fig 11). This shows that the subset of virulence-related genes used are not enough to determine genetic differences and to explain differences in virulence between the strains.

We conclude that the predictive value of different model systems varies since the relative virulence across fungal strains does not hold up across different infection models. We propose that differences observed in the four infection models mostly depends on the genetic make-up, and maybe also epi-genetic changes, of each *A*. *fumigatus* strains. Each infection system delivered its own order of virulence of the strains which is a reflection of virulence traits of the fungus in the context of each specific host. Furthermore, characteristics of conidial germination can affect the outcome in infections models. Comparison of infection models is therefor difficult as multiple factors of pathogen and host influence the final outcome the infection.

## Materials and methods

### Strains and growth conditions

Strains used in this study (Table 3) were grown for 3 days on potato dextrose agar (PDA) (Difco) at 37°C. Conidia were harvested with 0.85% (w/v) NaCl with or without Tween 20 (0.005%) (VWR international) depending on the type of experiments and filtered through 3 layers of sterile Miracloth (Merck Millipore) to remove mycelium and hyphae. Conidia were counted using a Bürker counting chamber.

**Table 3:** Strains used in this study.

Strain	Description	Origin	Reference
Af293.1	pRG3AMA1-RFP	Human, invasive infection	[60]
CEA10	Wild type	Human, invasive infection	[61]
ATCC46645	Wild type	Human, invasive infection	ATCC*
DTO271-B5	Wild type	Canine, non-invasive infection	[47]
DTO303-F3	Wild type	Canine, non-invasive infection	[47]

*the strain was kindly provided by Prof. Dr. Axel A. Brakhage from HKI, Jena, Germany.

### Peroxide and menadione sensitivity assays

Conidia were harvested in 0.85% (w/v) NaCl with 0.005% Tween 20. Peroxide and menadione sensitivity assays were performed in agar diffusion assays as described previously [62,63]. Briefly 5 10^7^ conidia were mixed in 5 mL minimal medium (MM; 6 g L^-1^ NaNO_3_, 1.5 g L^-1^ KH_2_PO_4_, 0.5 g L^-1^ KCl, 0.5 g L^-1^ MgSO_4_·7H_2_O, 0.2 mL L^-1^ Vishniac) with 1% agar and poured onto MM agar plates (6 cm in diameter with 10 mL MM) with 20 mM glucose (Sigma Aldrich). A 10 mm-wide hole was punched in the middle of the plate and filled with 100 μL 500 mM H_2_O_2_ (Sigma Aldrich) or 100 μL 1 mM menadione (Sigma Aldrich). Plates were incubated at 37°C for 16 hours after which the inhibition zone was measured.

### Size of conidia

The size of conidia was determined as described [37]. In short, conidia were diluted to an amount of 10^5^ conidia mL^-1^ ISOTON II diluent (Beckman Coulter). 100 μL conidia solution was used to determine the size distributions using a Beckman Coulter Counter Multisizer 3 equipped with a 70-μm aperture tube. 10^3^ datapoints were extracted and used for analysis. Objects with a diameter < 2 and > 6 μm were excluded from the dataset.

### Germination of conidia and mycelium growth

Germtube formation was quantified after swelling conidia for 4, 6 or 8 h in Czapek-Dox medium (CZD) (Difco) at 37°C. For each time-point and strain at least 1000 conidia or germlings were counted.

Mycelium growth was measured as described by [47]. In short, conidia were 3-point inoculated on PDA plates and incubated lid-side up at 37°C for 2 days. Diameter of the colonies was measured using the Fiji image processing package of ImageJ (www.fiji.sc)

### Cell culture and fungal infection

The human lung carcinoma epithelial cell line A549 (ATCC, CCL-185) was maintained by serial passage in Dulbecco’s modified Eagle medium (DMEM) (Ref. code: 11995–065, Gibco) supplemented with 10% fetal bovine serum (FBS) (Gibco). Fungal infections were performed as described [64]. In short, cells were seeded at a concentration of 2 10^5^ cells mL^-1^ and cultured at 37°C with 5% CO_2_ until a confluent monolayer was formed. Cells in 12, 24 or 48 wells plates (Corning®, Costar®) were challenged with 2 10^6^ conidia mL^-1^ DMEM + 10% FBS, resulting in a multiplicity of infection (MOI) of 1. Cells were cultured in 48 wells plates containing 8 mm glass coverslips (ThermoFisher Scientific) for internalization, association and germination experiments, in 12 wells plates for the IL-8 secretion or in 24 wells plates containing 12 mm glass coverslips (VWR International) for the identification of apoptotic and necrotic cells (see below).

### Internalization, association and germination of conidia with A549 cells

Internalization and association experiments were performed as described [64]. In short, A549 lung epithelial cells were grown on 8 mm glass coverslips (ThermoFisher Scientific) until a confluent layer was formed. Conidia of CEA10, ATCC46645, DTO271-B5 and DTO303-F3, which did not express a fluorescent protein such as *RFP* in Af293.1, were stained with 20 μg mL^-1^ *Aspergillus* FITC labelled antibody (ThermoFisher Scientific) for 60 min at room temperature before addition to the A549 cells. Unbound antibody was not removed by washing to avoid loss of conidia during pelleting. Conidia were added to A549 cells at an MOI of 1 and incubated for 2 h. Unbound conidia were removed by washing three times with pre-warmed DMEM + 10% FBS and incubation was continued for 2 h, resulting in a total infection time of 4 h. Conidia adhering to the A549 cells were visualized with 1% calcofluor white (CFW) (Sigma Aldrich) in DMEM + 10% FBS. CFW was added for 10 min at 37°C followed by one washing step with DMEM + 10% FBS. The cells and conidia were fixed with 4% paraformaldehyde (PFA) (VWR international) for 5 min at 4°C and 20 min at room temperature. PFA background fluorescence was quenched by a 20 min incubation at room temperature with 20 mM NH_4_Cl (Acros Organics). A549 cells were visualized with 1 μg mL^-1^ Hoechst (BD Biosciences). Coverslips were mounted to a glass slide using FluorSave^tm^ (Merck Millipore) and dried overnight. The number of conidia associated to the A549 cells (number of conidia per A549 cell) was determined by analysing 10 randomly chosen fields at the coverslip for imaging. Internalization of the conidia was determined by analysing z-stacks made at 10 randomly chosen fields of the coverslip. Conidia which were red or green were counted as internalized, whereas conidia that also stained with CFW were classified as adhering non-internalized conidia since CFW is not able to penetrate into the A549 cells. Internalization values were expressed as the percentage of the total conidia that associated with the cells. Experiments were performed as biological triplicates, with pictures taken at 10 random places on the slide. At least 100 conidia were counted per strain in each replicate.

Germination experiments were based on experiments described [20]. A549 cells were grown on 8 mm glass coverslips (ThemoFisher Scientific) until a confluent layer was formed. Conidia were added to the A549 cells at an MOI of 1 and incubated for 2 h. Unbound conidia were then removed by washing three times with pre-warmed DMEM + 10% FBS. Incubation then continued for another 10 h, making the total incubation time of the conidia with the A549 cells 12 h. Associated conidia and hyphae were stained with 1% CFW in DMEM + 10% FBS for 10 min at 37°C followed by one washing step with pre-warmed DMEM + 10% FBS. Cells and conidia were then fixed with 4% PFA for 5 min at 4°C and 20 min at room temperature. PFA background fluorescence was quenched by a 20 min incubation at room temperature with 20 mM NH_4_Cl. Conidia and hyphae that did not express a reporter protein (CEA10, ATCC, DTO271-B5 and DTO303-F3), such as *RFP* in Af293.1, were visualized using antibodies. To this end the cells were first blocked by a 1 h incubation with 0.3% bovine serum albumin (BSA) (Sigma Aldrich) in PBS. The primary anti-*Aspergillus* antibody (Rabbit anti-*Aspergillus*, ab20419 Abcam) was 1000-fold diluted in PBS and incubated with the cells for 2 h. Cells were washed once with PBS, after which the secondary antibody (Goat anti-Rabbit AlexaFluor 488, #4412 Cell Signaling Technology), which had been 1000-fold diluted in PBS, was added to the cells for 1 h. Coverslips were mounted to the glass slide using FluorSave^tm^ and dried overnight. The percentage of germinated conidia was determined by analysing z-stacks made at 10 randomly chosen fields on the coverslip. Hyphae and conidia that were green or red and stained with CFW were counted as outside of the A549 cells. Germination values were expressed as the percentage of germinated conidia from all associated conidia to the A549 cells. Biological triplicates were used with pictures taken at 10 random places on the slide. At least 100 conidia or hyphae were counted in each replicate.

### Quantification of IL-8 secretion

Confluent cell layers of A549 cells were challenged in 12 wells plates (Corning®, Costar®) with conidia for 2 h, after which the unbound conidia were removed by washing three times with pre-warmed DMEM. Exposure of the A549 cells to the conidia was continued for another 2 or 10 h. The culture medium was added to 96 wells IL-8 ELISA plates (ThermoFisher Scientific) according to the manufacturer’s instructions. Experiments were performed using biological triplicates and technical duplicates.

### Identification of apoptotic and necrotic cells

A549 cells were grown on 12-mm glass coverslips (VWR international) until a confluent cell layer was formed. The A549 cells were challenged with conidia for 2 h, after which unbound conidia were removed by washing three times with pre-warmed DMEM + 10% FBS. Exposure of the A549 cells to the conidia continued for another 2 or 10 h and washed twice with PBS. Staining of the apoptotic and necrotic cells was based on [65]. In short, 100 μg mL^-1^ acridine orange (AO) (VWR International) and 100 μg mL^-1^ ethidium bromide (EB) (Sigma Aldrich) was added to PBS for each experiment and added to the cells. The cells were immediately mounted on a coverslip with FluorSave^tm^ and examined by fluorescence microscopy within 15 min after adding the dye. The experiments were performed using biological triplicates with pictures taken at 10 random places on the slide. At least 100 cells per strain were analysed per replicate.

### A549 cell damage

Confluent layers of A549 cells in 24 wells plates (Corning®, Costar®) were challenged with conidia for 2 h, after which unbound conidia were removed by washing three times with pre-warmed DMEM. Exposure of the A549 lung cells to the conidia was continued for another 2 or 10 h. Cell damage at both time points of challenging (4 and 12 h in total) was measured by lactate dehydrogenase (LDH) released into the medium. The medium was added to a transparent 96 wells plate (Corning®, Costar®) and LDH activity was measured using an LDH activity kit (Sigma Aldrich) according to manufacturer’s instructions. A549 cells not challenged with conidia served as control. Experiments were done using biological triplicates and technical duplicates.

### *Galleria melonella* infection experiments

*Galleria melonella* larvae were injected in the hindleg with 10 μL PBS containing 10^5^, 10^6^ or 10^7^ conidia mL^-1^ and placed in the dark in a 37°C incubator. Survival of the larvae was monitored for 7 days after infection. Larval death was assessed by the lack of movement and the presence of a black pigment in the larvae. Fungal survival was monitored for 3 days after infection. At day 0, 1, 2 and 3 the haemolymph of three larvae for each condition was collected in an Eppendorf tube by bleeding. The haemolymph was homogenized and diluted in PBS and plated onto PDA agar plates containing 50 μg mL^-1^ ampicillin (Sigma Aldrich) to prevent bacterial growth. Plates were incubated overnight at 37°C and fungal survival was determined by the colony forming units (CFU). All experiments were done using biological triplicates.

### *Protostelium aurantium* growth and fungal infection

*Protostelium aurantium* var. *fungivorum* was grown as described [27] at 22°C in Petri dishes with phosphate buffer (PB: 26.8 g L^-1^ Na_2_HPO_4_∙7H_2_O, 2.4 g L^-1^ KH_2_PO_4_, pH 6.4) and with the pigmented yeast *Rhodotorula mucilaginosa* as food source. Fungal survival and phagocytosis by the amoebae were determined as described [27,39]. For fungal survival the conidia were allowed to swell in CZD for 4, 6 or 8 h at 37°C. *P*. *aurantium* cells were washed three times with PB to remove residual *R*. *mucilaginosa* and added to the swollen conidia at an MOI of 10 and incubated for 18 h at 22°C. Subsequently, the plates were transferred to 37°C for 1 h to kill the amoeba and 0.002% (w/v) resazurin (Sigma Aldrich) was added to the plate. Fungal survival was expressed as the percentage of resorufin formation by fungi confronted with *P*. *aurantium* and the resorufin formation by fungi alone. Resorufin fluorescence was measured after 15 h of incubation at 37°C with an Infinite M200 Pro fluorescence plate reader (Tecan). Experiments were done using biological and technical triplicates.

For phagocytosis of conidia by amoeba, conidia were first stained for 30 min with 0.1 mg mL^-1^ FITC (Sigma Aldrich) in 0.1 M Na_2_CO_3_ on a rotary shaker at 50 rpm at room temperature. Excess FITC was removed by washing three times with PBS. Conidia were allowed to swell for 4.5 h in CZD at 37°C. *P*. *aurantium* cells were treated as above, added to the swollen conidia at an MOI of 10 and incubated at 22°C for 2 h. The growth medium was aspirated and replaced by 0.1 mg mL^-1^ CFW (fluorescent brightener 28) (Sigma Aldrich) in PBS. Excess CFW was removed by washing once with PBS. Amoeba and fungi were fixed using 3.7% formaldehyde (Carl Roth) for 10 min prior to imaging. Experiments were done using three biological replicates, with at least 100 amoebae counted for each replicate.

To study germination of conidia after incubation with amoebae, conidia were stained for 30 min with 0.1 mg mL^-1^ FITC in 0.1 M Na_2_CO_3_ on a rotary shaker at 50 rpm at room temperature. Excess FITC was removed by washing three times with PBS. Conidia were allowed to swell for 4, 6 or 8 h in CZD at 37°C. *P*. *aurantium* cells were added to the swollen conidia at an MOI of 10 and incubated for 18 h at 22°C. Fixation with formaldehyde and staining with CFW was carried out as above. Experiments were done using three biological replicates, and at least 100 conidia were counted for each replicate. Hyphal length was measured in μM using the Fiji image processing package of ImageJ (www.fiji.sc).

### Zebrafish care and maintenance and infection experiments

Wild-type AB/TL zebrafish lines, adults and embryos, were handled in compliance with the local animal welfare regulations and maintained according to standard protocols (zfin.org). Breeding of the zebrafish adults was approved by the local animal welfare committee (DEC) of the University of Leiden under license number 10612 and in accordance with the international guidelines specified by the EU Animal Protective Directive 2010/63/EU. All zebrafish studies were performed on embryos before the free feeding stage and therefore the work did not fall under the animal experimentation law according to the EU Animal Protection Directive 2010/63/EU. Adult zebrafish were kept at 28°C in an aquarium with a 14 h day and 10 h night cycle. Embryos were cultured at 28.5°C in egg water (60 μg mL^-1^ sea salt; Seramarin) and were anesthetized in the same solution containing 0.02% (w/v) buffered Tricaine (3-aminobenzoic acid ethyl ester) (Sigma Aldrich) before fungal microinjections.

Fungal microinjection into the hindbrain of the zebrafish was done as described [32]. In short, conidia (10^8^ conidia mL^-1^) were taken up in PBS with 2% polyvinylpyrrolidone (PVP). The injected dosage of approximately 100 conidia per larvae was microscopically checked at 40 times magnification. The conidia were injected into the hindbrain ventricle of zebrafish embryos via the otic vesicle at 36 h post fertilization (HPF). The survival of embryos was monitored until 5 days post fertilization (DPF). Experiments were done using biological triplicates, for each replicate 48 zebrafish were injected per strain. Zebrafish were scored as dead when there was no observable heartbeat.

### Microscopy

Confocal images of internalized, associated and germinated conidia upon interaction with A549 lung epithelial cells were acquired with a Zeiss LSM 700 microscope using the Plan-Apochromat 63 x 1.40 oil DIC (WD = 0.19) objective. Images were taken using the 405, 488 and 555 nm laser lines. Fluorescence emission of CFW and Hoechst was detected using the 400–490 nm spectral band. Red fluorescence emission of RFP was detected with the 560–700 nm spectral band and FITC fluorescence was detected with the 490–555 nm spectral bands.

Images of the apoptotic and necrotic cells were acquired with an Axioskop 2 plus at a 20 x magnification. The FITC filter with an excitation of 450–488 nm was used to image Acridine Orange with an excitation of 431 nm and the TRITC filter with an excitation of 540–570 nm was used to image the ethidium bromide with an excitation of 524 nm. Phagocytosis of the conidia by amoeba was visualized using a Zeiss Axio Observer 7 spinning disk confocal microscope using the 63 x oil objective. Images were taken using the bright field to image the amoeba, the 405 nm laser line was used to image the FITC stained conidia and the 488 nm laser line was used to image the CFW stained conidia.

All images were analysed and processed with the Fiji image processing package of ImageJ (www.fiji.sc).

### Chromosomal DNA isolation

Conidia were inoculated in transformation medium (TM; MM supplemented with 5 g L^-1^ yeast extract (Difco) and 2 g L^-1^ casamino acids (Difco)) and 50 μg mL^-1^ ampicillin (Sigma Aldrich), to prevent bacterial contamination, and grown overnight at 37°C and 200 rpm. Mycelium was collected by filtering over a double layer of Miracloth (Merck Millipore) and lyophilized overnight. Part of the lyophilized mycelium (~30 mg) was lyzed with a Tissuelyser (Qiagen) using 2 metal balls (4.76 mm in diameter) for 2 minutes at 25 Hz. DNA was isolated from the homogenized mycelium with the Qiagen DNeasy PowerPlant Pro kit following the manufacturer’s protocol for problematic samples. Qubit® was used to check DNA quality and concentration. DNA samples were stored at -20°C.

### Whole genome sequencing and virulence-related genes

DNA sequencing of strains ATCC46645 and CEA10 was performed by the Utrecht sequencing facility (Useq). Libraries were prepared using Truseq DNA Nano library and sequenced on an Illumina NextSeq500 with 150 bp pair end mid output configuration. Read quality was checked using FastQC (https://www.bioinformatics.babraham.ac.uk/projects/fastqc/), cleaning and trimming was performed with the Fastx-Toolkit (http://hannonlab.cshl.edu/fastx_toolkit/). The genome sequence of Af293 (version 42 from FungiDB https://fungidb.org/fungidb/) was used as the reference to map reads using bowtie2 v2.2.9 [66]. Freebayes v0.9.10–3 [67] was used for variant calling. Virulence-related genes have been described previously [41]. Custom R scripts were used to find and filter the SNPs in these genes, effects of the SNPs were predicted using SnpEff v3.3k [42].

### Phylogenetic tree

A set of published *A*. *fumigatus* genomes (S4 Table) was used. Alignment and variant calling of the genomes were performed as described above. The final set of variants of the strains used in this study and those described in S4 Table were used to build a SNP-based tree as described [68]. In short, SNPRelate V3.7 [69] was used to filter biallelic SNPs with no missing data, a linkage disequilibrium threshold of 0.8 and a minor allele frequency of 0.03. In total 14100 SNP markers were aligned with MAFFT 7.310 using default options [70]. RaxML was used to obtain a maximum likelihood tree with the GTRGAMMA model with 1000 bootstrap replicates [71]. The best-scoring ML tree was used for analysis. Figtree was used to visualize and analyze the phylogenetic tree [72].

### Statistical analysis

A one-way ANOVA with a p-value ≤ 0.05, with a Tukey test with a Bonferroni correction for multiple testing as a *post hoc* test, was used to analyse differences in inhibition zones after peroxide or menadione exposure, conidial association to A549 cells, amount of IL-8 secretion, LDH release and hyphal length after co-incubation with A549 cells or amoeba. The non-parametric Kruskal-Wallis test, with a pairwise Wilcoxon rank sum test with a Bonferroni correction for multiple testing as a *post hoc* test, was used to analyse differences in internalization and survival of conidia after incubation with the amoeba or after injection in the *G*. *melonella* larvae with a P-value ≤ 0.05 considered significant. The same test was used to assess the number of amoebae with ingested conidia, the germination of the conidia after co-incubation with the amoeba and amoeba survival after co-incubation with conidia. Survival of the *G*. *melonella* larvae and zebrafish embryos was plotted using the Kaplan-Meier survival curve. Differences in survival between strains was determined using a non-parametric log rank test. P-values ≤ 0.05 were considered significant.

## Supporting information

S1 Fig Relative abundance of the diameter (μm) of the conidia measured with the coulter counter, dashed line represents the mean of all the measured particles. (DOCX)

S2 Fig Representative images of association (A and B), conidia are shown in red (A) and A459 cells are stained with Hoechst and shown in blue(B), and internalization (C and D), conidia are shown in red (C) and conidia also stained with CalcoFluor White (CFW) in blue (D) are considered to be outside of the A549 cells, after 4 h of incubation.
(DOCX)

S3 Fig Representative figure of germination of conidia. Conidia and hyphae are shown in red (A) and conidia and hyphae outside of the A549 cells are shown in blue by CFW staining (B) after 12 hours of incubation with A549 cells.
(DOCX)

S4 Fig Representative image of the cells after infection with *A*. *fumigatus* conidia and dual acridine orange and ethidium bromide staining. Green cells represent cells that are alive (L), where the acridine orange can bind the double stranded DNA. Orange cells are apoptotic cells (A), where the acridine orange can bind the RNA or single stranded DNA. Red cells are cells where the ethidium bromide can enter indicating that they are necrotic (N).
(DOCX)

S1 Table Virulent-related genes as described by [41], with the type of SNP (low, moderate or high impact) indicated per strain. (DOCX)

S2 Table Shared SNPs with the location and amino acid change in *fma-PKS* (Afu8g00370) in the ATCC46645, CEA10, DTO271-B5 and DTO303-F3 strains. (DOCX)

S3 Table Base pair (BP) change, location and amino acid (AA) substitution in the genes with SNPs shared between ATCC46645 and DTO303-F3. Shared SNPs are displayed in bold.
(DOCX)

S4 Table Strains used for the assembly of the phylogenetic tree. (DOCX)

S5 Table Summary of comparison of *A*. *fumigatus* strains. Table is based upon data in the figures of the main in the main paper, used statistics can be found there and in the material and methods section.
(DOCX)

S6 Table Base pair (BP) change, location and amino acid (AA) substitution in the *FtmD* (Afu8g00200) gene. The SNPs described by [58] is highlighted in bold.
(DOCX)

S1 File (DOCX)
