# Correction: Variation of virulence of five *Aspergillus fumigatus* isolates in four different infection models

**DOI:** 10.1371/journal.pone.0258072

**Published:** 2021-09-27

**Authors:** E. M. Keizer, I. D. Valdes, G Forn-Cuni, E. Klijn, A. H. Meijer, F. Hillmann, H. A. B. Wösten, H. de Cock

The sixth author’s name is spelled incorrectly. The correct name is: F. Hillmann. The correct citation is: Keizer EM, Valdes ID, Forn-Cuni G, Klijn E, Meijer AH, Hillmann F, et al. (2021) Variation of virulence of five *Aspergillus fumigatus* isolates in four different infection models. PLoS ONE 16(7): e0252948. https://doi.org/10.1371/journal.pone.0252948
