# Supplementary material for: Variation of virulence of five Aspergillus fumigatus isolates in four different infection models
Source: PLoS One. 2021 Jul 9;16(7):e0252948. doi: 10.1371/journal.pone.0252948 (PMC8270121; doi:10.1371/journal.pone.0252948)
Supplement: S1 Fig — (DOCX) [file pone.0252948.s001.docx]

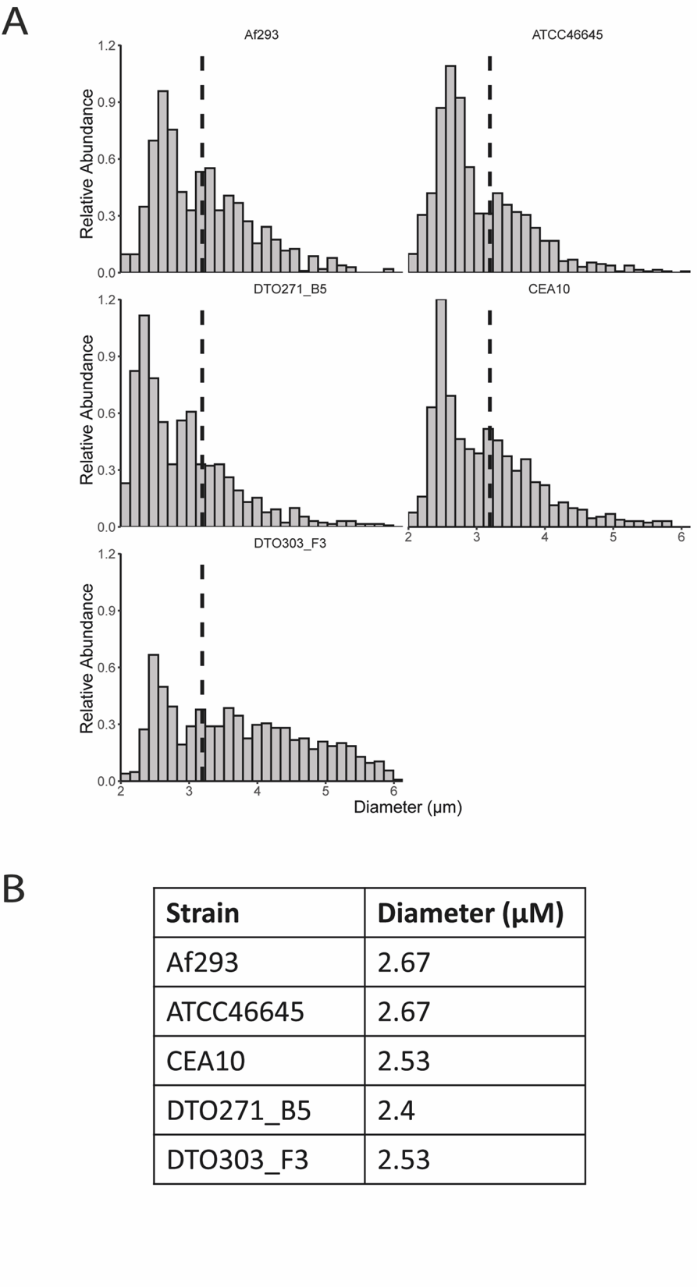


**Supplementary figure 1.** Relative abundance of the diameter (µm) of the conidia measured with the coulter counter, dashed line represents the mean of all the measured particles.
