# Supplementary material for: Variation of virulence of five Aspergillus fumigatus isolates in four different infection models
Source: PLoS One. 2021 Jul 9;16(7):e0252948. doi: 10.1371/journal.pone.0252948 (PMC8270121; doi:10.1371/journal.pone.0252948)
Supplement: S2 Fig — Representative images of association (A and B), conidia are shown in red (A) and A459 cells are stained with Hoechst and shown in blue(B), and internalization (C and D), conidia are shown in red (C) and conidia also stained with CalcoFluor White (CFW) in blue (D) are considered to be outside of the A549 cells, after 4 h of incubation. (DOCX) [file pone.0252948.s002.docx]

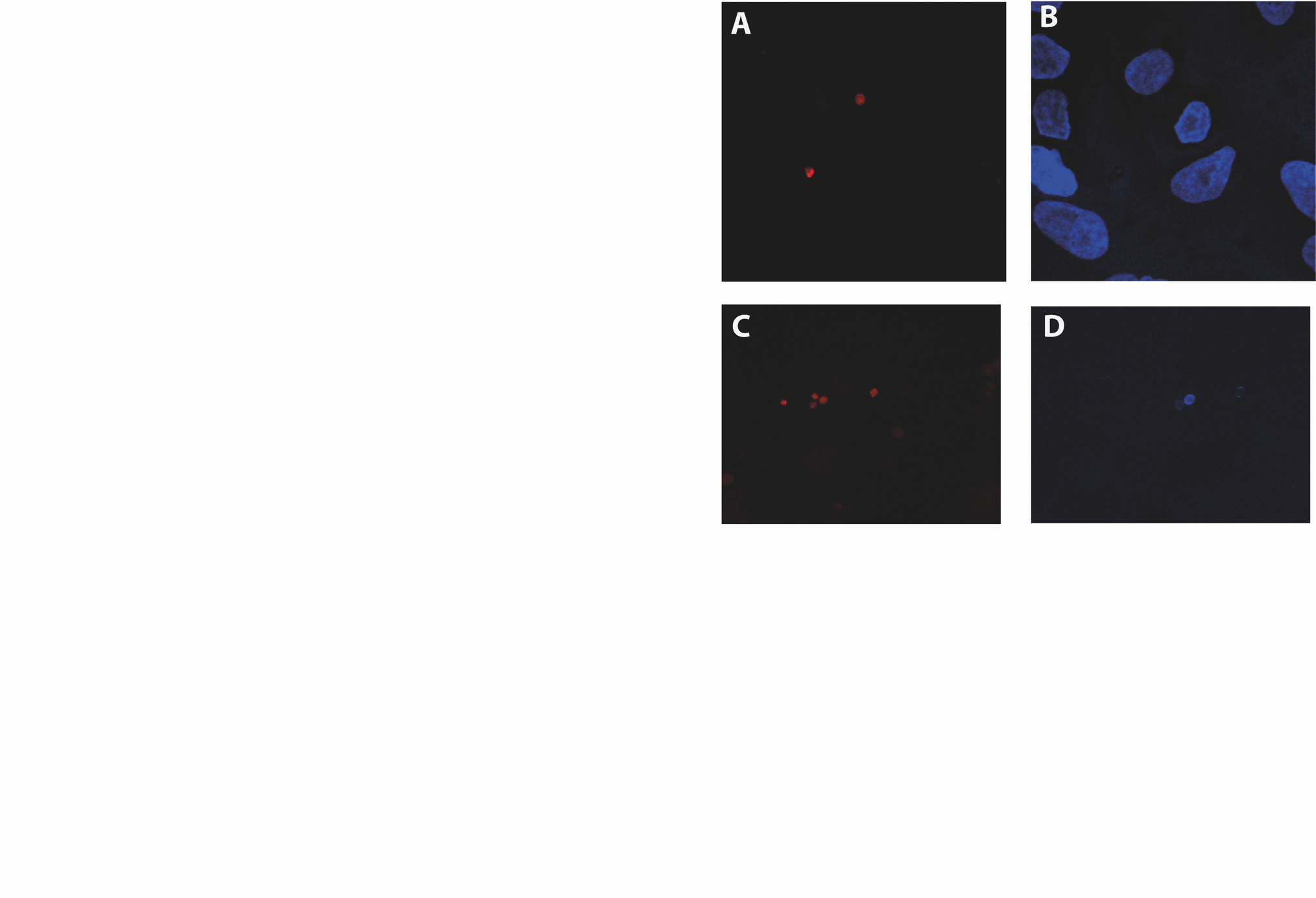


**Supplementary figure 2.** Representative images of association (A and B), conidia are shown in red (A) and A459 cells are stained with Hoechst and shown in blue(B), and internalization (C and D), conidia are shown in red (C) and conidia also stained with CalcoFluor White (CFW) in blue (D) are considered to be outside of the A549 cells, after 4 h of incubation.
