# Supplementary material for: Variation of virulence of five Aspergillus fumigatus isolates in four different infection models
Source: PLoS One. 2021 Jul 9;16(7):e0252948. doi: 10.1371/journal.pone.0252948 (PMC8270121; doi:10.1371/journal.pone.0252948)
Supplement: S3 Fig — Conidia and hyphae are shown in red (A) and conidia and hyphae outside of the A549 cells are shown in blue by CFW staining (B) after 12 hours of incubation with A549 cells. (DOCX) [file pone.0252948.s003.docx]

**
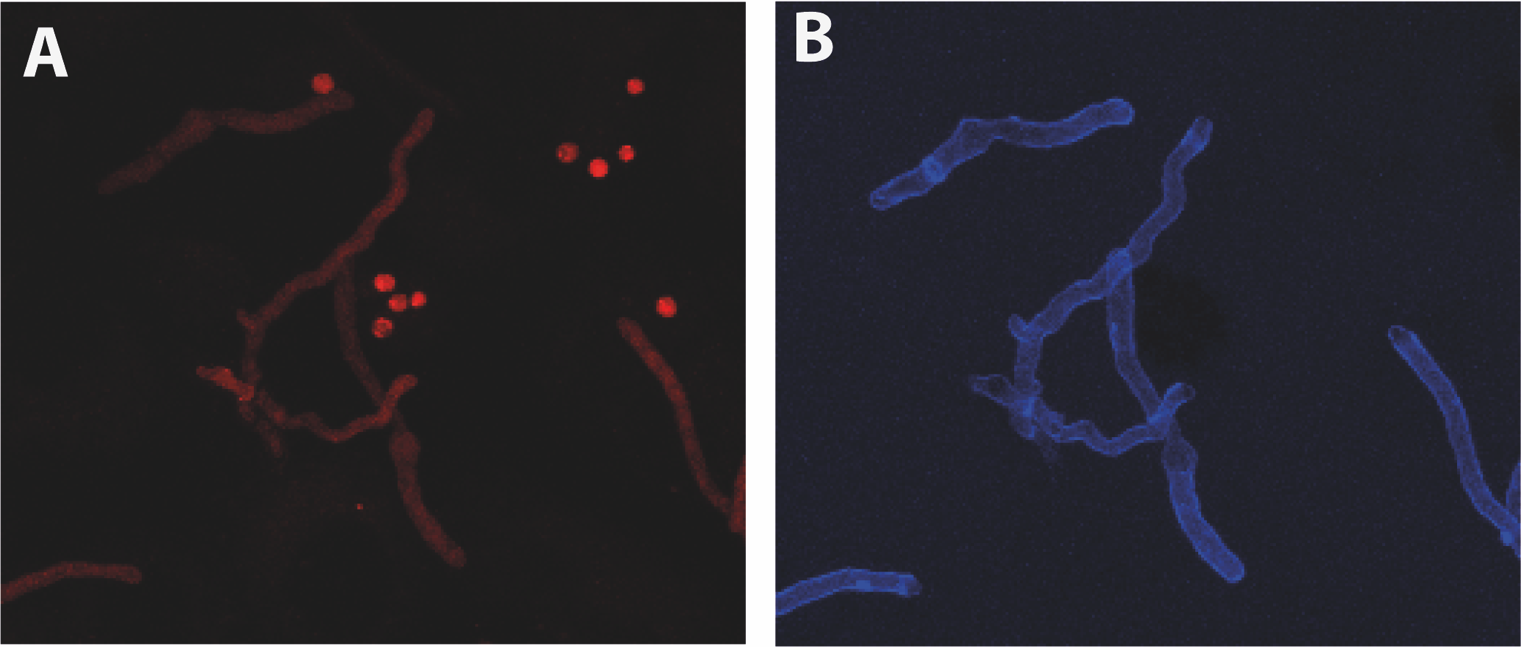
**

**Supplementary figure 3.** Representative figure of germination of conidia. Conidia and hyphae are shown in red (A) and conidia and hyphae outside of the A549 cells are shown in blue by CFW staining (B) after 12 hours of incubation with A549 cells.
