# Supplementary material for: Variation of virulence of five Aspergillus fumigatus isolates in four different infection models
Source: PLoS One. 2021 Jul 9;16(7):e0252948. doi: 10.1371/journal.pone.0252948 (PMC8270121; doi:10.1371/journal.pone.0252948)
Supplement: S4 Fig — Green cells represent cells that are alive (L), where the acridine orange can bind the double stranded DNA. Orange cells are apoptotic cells (A), where the acridine orange can bind the RNA or single stranded DNA. Red cells are cells where the ethidium bromide can enter indicating that they are necrotic (N). (DOCX) [file pone.0252948.s004.docx]

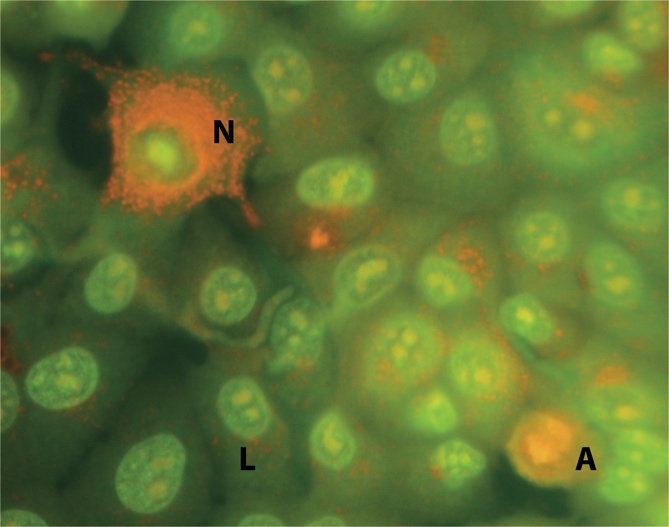


**Supplementary figure 4.** Representative image of the cells after infection with *A. fumigatus* conidia and dual acridine orange and ethidium bromide staining. Green cells represent cells that are alive (L), where the acridine orange can bind the double stranded DNA. Orange cells are apoptotic cells (A), where the acridine orange can bind the RNA or single stranded DNA. Red cells are cells where the ethidium bromide can enter indicating that they are necrotic (N).
