# Supplementary material for: Variation of virulence of five Aspergillus fumigatus isolates in four different infection models
Source: PLoS One. 2021 Jul 9;16(7):e0252948. doi: 10.1371/journal.pone.0252948 (PMC8270121; doi:10.1371/journal.pone.0252948)
Supplement: S1 Table — (DOCX) [file pone.0252948.s005.docx]

**Supplementary Table 1.** Virulent-related genes as described by [1], with the type of SNP (low, moderate or high impact] indicated per strain.

| **Function** | **Gene ID** | **Gene Name** | **ATCC46645** | |  | **CEA10** |  |  | **DTO271-B5** | |  | **DTO303-F3** | |  |
| --- | --- | --- | --- | --- | --- | --- | --- | --- | --- | --- | --- | --- | --- | --- |
|  |  |  | **low** | **moderate** | **high** | **low** | **moderate** | **high** | **low** | **moderate** | **high** | **low** | **moderate** | **high** |
| Thermotolerance | Afu1g03992 | thtA | x |  |  | x | x |  | x | x |  | x |  |  |
|  | Afu3g06450 | pmt1 | x |  |  |  |  |  | x |  |  | x |  |  |
|  | Afu5g04170 | hsp90 |  |  |  |  |  |  |  |  |  |  |  |  |
|  | Afu8g02750 | cgrA |  |  |  |  |  |  |  |  |  |  |  |  |
| Resistance to immune response | Afu1g03200 | mfsC | x | x |  | x | x |  | x | x |  | x | x |  |
|  | Afu1g10380 | nrps1 | x | x |  | x | x |  | x | x |  |  |  |  |
|  | Afu1g10390 | abcB | x | x |  | x |  |  | x |  |  |  |  |  |
|  | Afu1g12690 | mdr4 |  |  |  |  |  |  |  |  |  |  |  |  |
|  | Afu1g13330 | arp2 |  |  |  |  |  |  |  |  |  |  |  |  |
|  | Afu1g14330 | abcC |  |  |  |  |  |  |  |  |  |  |  |  |
|  | Afu1g14550 | sod3 |  |  |  | x |  |  |  |  |  |  |  |  |
|  | Afu1g15490 | mfsB | x |  |  | x | x |  | x | x |  | x |  |  |
|  | Afu1g17250 | rodB | x |  |  |  |  |  |  |  |  |  |  |  |
|  | Afu1g17440 | abcA |  | x |  | x | x |  | x | x |  | x | x |  |
|  | Afu2g17530 | abr2 | x |  |  | x |  |  | x | x |  | x | x |  |
|  | Afu2g17550 | ayg1 | x | x |  | x | x |  | x | x |  |  | x |  |
|  | Afu2g17600 | pksP | x | x |  |  | x |  | x |  |  | x | x |  |
|  | Afu3g02270 | cat1 |  |  |  | x |  |  | x |  |  |  |  |  |
|  | Afu3g03500 | mdr3 | x | x |  | x | x |  | x | x |  | x | x |  |
|  | Afu3g09690 | catA |  | x |  |  | x |  | x | x |  |  |  |  |
|  | Afu3g10830 | gstA | x |  |  | x |  |  | x |  |  | x |  |  |
|  | Afu3g12120 | ppoC | x | x |  | x | x |  | x | x |  | x | x |  |
|  | Afu4g00180 | ppoB | x |  |  | x |  |  | x |  |  | x |  |  |
|  | Afu4g10000 | mdr2 | x | x |  | x | x |  | x | x |  |  | x |  |
|  | Afu4g10770 | ppoA | x | x |  |  | x |  |  | x |  |  | x |  |
|  | Afu4g11580 | sod2 |  |  |  |  |  |  |  |  |  |  |  |  |
|  | Afu4g13390 | arpA |  |  |  |  |  |  |  |  |  |  |  |  |
|  | Afu4g14530 | tpcF | x | x |  | x | x |  | x | x |  |  |  |  |
|  | Afu5g06070 | mdr1 |  | x |  | x | x |  | x | x |  | x | x |  |
|  | Afu5g09240 | sod1 |  |  |  |  |  |  |  |  |  |  |  |  |
|  | Afu5g09580 | rodA | x | x |  | x | x |  |  |  |  |  |  |  |
|  | Afu6g03470 | fmpD | x | x |  | x | x |  |  |  |  | x | x |  |
|  | Afu6g03890 | catA | x |  |  | x |  |  | x | x |  | x |  |  |
|  | Afu6g04360 | atrF | x | x |  | x | x |  | x | x |  | x | x |  |
|  | Afu6g07210 | sod4 | x |  |  |  |  |  |  |  |  | x |  |  |
|  | Afu6g09930 | yap1 | x | x |  | x | x |  | x |  |  |  |  |  |
|  | Afu6g12522 | skn7 |  |  |  |  | x |  |  |  |  |  |  |  |
|  | Afu7g00480 | abcE | x | x |  | x | x |  | x | x |  | x | x |  |
|  | Afu7g05500 | gstB |  |  |  | x |  |  | x |  |  | x | x |  |
|  | Afu8g01670 | cat2 | x |  |  | x |  |  | x |  |  | x |  |  |
|  | Afu8g05710 | mfsA | x | x |  | x | x |  | x | x |  | x | x |  |
| Cell wall | Afu1g01380 | och4 | x |  |  | x |  |  | x |  |  |  |  |  |
|  | Afu1g04260 | ENGL1 | x | x |  | x | x |  | x | x |  | x | x |  |
|  | Afu1g07690 | afpmt2 | x |  |  |  |  |  |  |  |  |  |  |  |
|  | Afu1g12600 | chsD | x | x |  |  | x |  | x | x |  |  |  |  |
|  | Afu1g13280 | pmi1 |  |  |  |  |  |  |  |  |  |  |  |  |
|  | Afu1g15440 | ags3 | x | x |  | x | x |  | x |  |  | x |  |  |
|  | Afu2g01170 | gel1 | x | x |  |  | x |  |  | x |  |  | x |  |
|  | Afu2g01450 | mnn9 |  |  |  |  | x |  |  |  |  |  | x |  |
|  | Afu2g01870 | chsA | x | x |  |  |  |  |  |  |  | x | x |  |
|  | Afu2g05150 | mp2 | x | x |  | x | x |  | x | x |  | x | x |  |
|  | Afu2g05340 | gel4 | x |  |  | x |  |  | x |  |  | x |  |  |
|  | Afu2g11270 | ags2 | x | x |  | x | x |  | x | x |  |  |  |  |
|  | Afu2g12850 | gel3 | x | x |  |  | x |  |  |  |  |  |  |  |
|  | Afu2g13440 | chsE | x | x |  | x | x |  | x | x |  | x | x |  |
|  | Afu2g15910 | anp1 |  |  |  |  |  |  |  |  |  |  |  |  |
|  | Afu2g17560 | arp2 | x |  |  |  |  |  | x | x |  |  |  |  |
|  | Afu2g17580 | arp1 |  |  |  |  |  |  |  |  |  |  |  |  |
|  | Afu3g00910 | ags1 | x | x |  | x | x |  | x | x |  | x | x |  |
|  | Afu3g06690 | rho3 |  |  |  |  |  |  |  |  |  |  |  |  |
|  | Afu3g10340 | rho2 |  |  |  |  |  |  |  |  |  |  |  |  |
|  | Afu3g12690 | glfA | x |  |  | x |  |  | x |  |  | x |  |  |
|  | Afu3g13200 | gel6 | x | x |  | x | x |  | x | x |  | x | x |  |
|  | Afu3g14420 | chsG | x | x |  |  |  |  |  |  |  | x |  |  |
|  | Afu4g03240 | mp1 | x |  |  | x |  |  | x |  |  | x |  |  |
|  | Afu4g04180 | chsB |  |  |  |  |  |  |  |  |  |  |  |  |
|  | Afu4g06820 | ecm33 | x |  |  |  | x |  |  |  |  |  | x |  |
|  | Afu5g00760 | chsC |  | x |  |  | x |  |  | x |  |  | x |  |
|  | Afu5g02740 | afmnt3 | x |  |  | x |  |  | x |  |  | x |  |  |
|  | Afu5g08580 | och1 | x | x |  | x | x |  | x | x |  | x | x |  |
|  | Afu5g10760 | mnt1 |  |  |  | x |  |  |  |  |  |  |  |  |
|  | Afu5g12160 | afmnt2 |  |  |  |  |  |  |  |  |  |  |  |  |
|  | Afu5g14060 | rho4 | x |  |  | x |  |  | x |  |  | x |  |  |
|  | Afu6g06900 | rho1 |  |  |  |  |  |  |  |  |  |  |  |  |
|  | Afu6g11390 | gel2 | x |  |  |  |  |  |  |  |  |  |  |  |
|  | Afu6g12400 | fks1 | x | x |  | x | x |  | x | x |  | x | x |  |
|  | Afu6g12410 | gel7 |  | x |  |  | x |  |  | x |  |  | x |  |
|  | Afu6g14040 | och2 | x |  |  | x | x |  | x |  |  | x | x |  |
|  | Afu8g02040 | och3 | x |  |  |  |  |  |  | x |  |  |  |  |
|  | Afu8g02130 | gel5 |  | x |  |  | x |  |  |  |  |  |  |  |
|  | Afu8g04500 | pmt4 | x |  |  | x |  |  | x |  |  | x | x |  |
|  | Afu8g05630 | chsF |  | x |  | x |  |  |  |  |  |  |  |  |
| Toxins and secondary metabolites | Afu1g14660 | laeA |  |  |  |  |  |  |  |  |  |  |  |  |
|  | Afu2g17540 | abr1 | x | x |  | x | x |  | x | x |  |  | x |  |
|  | Afu2g17970 | fgaFS | x | x |  |  |  |  |  |  |  |  | x |  |
|  | Afu2g17980 | easK | x | x |  |  |  |  | x |  |  |  |  |  |
|  | Afu2g18000 | fgaDH |  |  |  |  |  |  |  |  |  |  | x |  |
|  | Afu2g18010 | easM | x |  |  |  |  |  |  |  |  | x | x |  |
|  | Afu2g18020 | fgaAT | x |  |  |  |  |  |  |  |  | x | x |  |
|  | Afu2g18030 | fgaCat | x | x |  |  |  |  |  |  |  |  | x |  |
|  | Afu2g18040 | dmaW |  | x |  |  |  |  | x | x |  |  | x |  |
|  | Afu2g18050 | fgaOx1 |  |  |  |  |  |  |  | x |  | x | x |  |
|  | Afu2g18060 | fgaMT |  | x |  |  |  |  |  | x |  |  | x |  |
|  | Afu3g12900 | hasB | x | x |  | x |  |  | x |  |  | x |  |  |
|  | Afu3g12940 | hasF |  | x |  | x | x |  |  | x |  | x | x |  |
|  | Afu3g12950 | hasG | x | x |  | x | x |  |  | x |  |  | x |  |
|  | Afu4g10460 | hcsA | x |  |  |  |  |  |  |  |  |  |  |  |
|  | Afu4g14480 | tpcL |  |  |  |  |  |  |  |  |  |  |  |  |
|  | Afu4g14490 | tpcJ | x | x |  |  | x |  | x |  |  |  | x |  |
|  | Afu4g14500 | tpcI | x | x |  | x |  |  | x |  |  | x |  |  |
|  | Afu4g14520 | tpcG |  |  |  |  |  |  |  |  |  |  |  |  |
|  | Afu4g14540 | tpcE |  |  |  |  |  |  |  |  |  |  |  |  |
|  | Afu4g14570 | tpcB |  | x |  |  |  |  |  | x |  |  | x |  |
|  | Afu4g14580 | tpcA | x |  |  | x |  |  | x |  |  | x | x |  |
|  | Afu4g14770 | osc3 | x | x |  | x |  |  | x |  |  | x |  |  |
|  | Afu4g14780 | cyp5081A1 | x | x |  |  |  |  |  | x |  |  |  |  |
|  | Afu4g14790 | cyp5081B1 | x | x |  | x | x |  | x | x |  | x | x |  |
|  | Afu4g14800 | sdr1 | x |  |  |  |  |  |  | x |  |  |  |  |
|  | Afu4g14820 | null |  | x |  | x |  |  | x |  |  |  |  |  |
|  | Afu5g12710 | null | x | x |  | x | x |  | x | x |  | x | x |  |
|  | Afu5g12720 | null | x | x |  | x | x |  | x | x |  | x | x |  |
|  | Afu5g12750 | null |  | x |  |  |  |  |  |  |  |  |  |  |
|  | Afu5g12760 | null | x | x |  |  |  |  |  |  |  | x | x |  |
|  | Afu5g12770 | null |  |  |  |  |  |  |  |  |  | x |  |  |
|  | Afu5g12780 | null | x |  |  | x |  |  | x |  |  | x |  |  |
|  | Afu5g12790 | null | x |  |  |  |  |  |  |  |  |  |  |  |
|  | Afu6g09630 | gliZ |  | x |  | x |  |  | x |  |  | x | x |  |
|  | Afu6g09640 | gliI |  | x |  | x | x |  | x | x |  | x | x |  |
|  | Afu6g09660 | gliP | x | x |  | x | x |  | x | x |  | x | x |  |
|  | Afu6g09670 | gliC | x |  |  | x |  |  | x |  |  |  |  |  |
|  | Afu6g09690 | gliG |  |  |  |  |  |  |  |  |  |  |  |  |
|  | Afu6g09710 | gliA |  |  |  |  |  |  | x | x |  |  |  |  |
|  | Afu6g09720 | gliN | x |  |  |  |  |  | x |  |  |  |  |  |
|  | Afu6g09730 | gliF | x |  |  |  | x |  | x |  |  |  |  |  |
|  | Afu6g09740 | gliT |  |  |  |  |  |  |  |  |  |  |  |  |
|  | Afu8g00190 | ftmC | x | x |  |  |  |  |  |  |  |  |  |  |
|  | Afu8g00200 | ftmD | x | x |  | x | x |  | x | x |  | x | x |  |
|  | Afu8g00370 | fma-PKS | x | x |  | x | x |  | x | x |  | x | x |  |
|  | Afu8g00380 | fmaC |  |  |  | x |  |  | x |  |  |  |  |  |
|  | Afu8g00390 | fmaD |  | x |  |  |  |  |  | x |  |  | x |  |
|  | Afu8g00400 | null | x | x |  |  |  |  | x | x |  | x | x |  |
|  | Afu8g00410 | metAP |  | x |  |  | x |  |  | x |  |  | x |  |
|  | Afu8g00420 | fumR |  | x |  |  | x |  |  | x |  |  | x |  |
|  | Afu8g00430 | null |  | x |  |  |  |  |  |  |  |  |  |  |
|  | Afu8g00440 | psoF |  |  |  | x |  |  | x |  |  | x |  |  |
|  | Afu8g00460 | fpaI | x |  |  | x |  |  |  |  |  | x |  |  |
|  | Afu8g00470 | fmaE | x |  |  | x |  |  | x |  |  | x |  |  |
|  | Afu8g00490 | Fma-KR | x | x |  | x | x |  |  |  |  | x | x |  |
|  | Afu8g00500 | null | x | x |  | x | x |  | x | x |  | x | x |  |
|  | Afu8g00510 | fmaG |  | x |  | x |  |  |  | x |  | x | x |  |
|  |  |  |  |  |  |  |  |  |  |  |  |  |  |  |
|  | Afu8g00540 | nrps14 | x | x |  | x | x |  | x | x |  | x | x |  |
|  | Afu8g00550 | psoC |  | x |  |  |  |  |  |  |  |  |  |  |
|  | Afu8g00570 | null |  |  |  | x |  |  |  |  |  | x |  |  |
|  | Afu8g00580 | psoE |  | x |  |  | x |  |  |  |  |  | x |  |
|  | Afu8g00520 | fma-TC | x |  |  | x |  |  |  |  |  | x |  |  |
| Allergens | Afu1g05770 | exg12 | x | x |  |  | x |  |  | x |  |  | x |  |
|  | Afu1g06830 | aspf26 |  |  |  |  |  |  |  |  |  |  |  |  |
|  | Afu1g09470 | aspfAT | x |  |  |  |  |  |  |  |  |  |  |  |
|  | Afu1g14560 | msdS | x |  |  | x |  |  | x |  |  |  |  |  |
|  | Afu1g16190 | aspf9 |  | x |  |  | x |  |  | x |  |  | x |  |
|  | Afu2g00760 | aspfPL | x | x |  | x | x |  | x | x |  | x | x |  |
|  | Afu2g03720 | aspf11 | x |  |  | x |  |  | x |  |  | x |  |  |
|  | Afu2g03830 | aspf4 | x | x |  | x | x |  | x | x |  | x | x |  |
|  | Afu2g10100 | aspf8 | x |  |  | x |  |  | x |  |  | x |  |  |
|  | Afu2g11260 | luA | x |  |  | x | x |  | x |  |  |  |  |  |
|  | Afu2g11850 | aspf23 |  |  |  |  |  |  |  |  |  |  |  |  |
|  | Afu2g12630 | aspf13 |  |  |  |  |  |  |  |  |  |  |  |  |
|  | Afu2g15430 | AspfSXR | x |  |  |  |  |  |  |  |  |  |  |  |
|  | Afu3g00590 | aspHS | x | x |  |  |  |  |  |  |  | x | x |  |
|  | Afu3g07430 | aspf27 |  |  |  | x |  |  | x |  |  | x |  |  |
|  | Afu3g14680 | aspfLPL3 | x |  |  | x |  |  | x |  |  | x |  |  |
|  | Afu4g01290 | csn | x | x |  | x |  |  | x |  |  | x |  |  |
|  | Afu4g06670 | aspf7 | x |  |  | x |  |  | x |  |  | x |  |  |
|  | Afu4g09580 | aspf2 |  |  |  |  |  |  |  |  |  |  |  |  |
|  | Afu5g02330 | aspf1 | x | x |  | x | x |  | x | x |  | x | x |  |
|  | Afu5g03520 | aspfPUP |  | x |  | x |  |  | x |  |  |  |  |  |
|  | Afu5g11320 | aspf29 | x |  |  | x |  |  |  |  |  |  |  |  |
|  | Afu6g02280 | aspf3 |  |  |  | x |  |  | x |  |  |  |  |  |
|  | Afu6g03620 | mreA | x | x |  | x | x |  | x | x |  | x |  |  |
|  | Afu6g04920 | fdh | x | x |  | x | x |  | x | x |  | x | x |  |
|  | Afu6g06770 | aspf22 |  |  |  | x |  |  |  | x |  |  |  |  |
|  | Afu6g10300 | aspf28 |  |  |  |  |  |  |  |  |  |  |  |  |
|  | Afu7g05740 | null |  |  |  |  |  |  |  |  |  |  |  |  |
| Nutrient uptake | Afu1g01550 | zrfA | x | x |  | x | x |  | x | x |  | x | x |  |
|  | Afu1g09280 | ptcB |  |  |  |  | x |  |  |  |  |  |  |  |
|  | Afu1g10080 | zafA |  |  |  |  | x |  |  | x |  |  |  |  |
|  | Afu1g16950 | pig-a |  |  |  | x |  |  |  |  |  |  |  |  |
|  | Afu1g17200 | sidC | x | x |  | x | x |  | x | x |  | x | x |  |
|  | Afu2g03860 | zrfB | x |  |  | x |  |  | x |  |  | x |  |  |
|  | Afu2g04010 | tpsB | x | x |  | x | x |  | x | x |  | x | x |  |
|  | Afu2g05730 | mirC | x |  |  | x |  |  | x |  |  | x |  |  |
|  | Afu2g07680 | sidA | x |  |  |  |  |  |  |  |  |  |  |  |
|  | Afu2g08360 | pyrG |  |  |  |  |  |  |  |  |  |  |  |  |
|  | Afu2g09030 | dppV |  | x |  |  | x |  |  | x |  |  | x |  |
|  | Afu3g03400 | sidF |  |  |  |  |  |  |  |  |  |  |  |  |
|  | Afu3g03420 | sidD | x | x |  | x | x |  | x | x |  | x | x |  |
|  | Afu3g03640 | mirB | x |  |  | x |  |  | x |  |  | x |  |  |
|  | Afu3g03650 | sidG | x |  |  | x |  |  | x | x |  | x |  |  |
|  | Afu3g05650 | orlA | x | x |  | x |  |  | x |  |  | x |  |  |
|  | Afu3g09820 | dvrA |  |  |  |  |  |  |  |  |  |  |  |  |
|  | Afu3g11400 | pep2 |  |  |  |  |  |  |  |  |  |  | x |  |
|  | Afu3g11970 | pacC | x | x |  | x | x |  |  | x |  |  | x |  |
|  | Afu4g07040 | ctsD |  |  |  |  |  |  |  |  |  |  |  |  |
|  | Afu4g08720 | plb1 |  |  |  | x |  |  |  |  |  |  |  |  |
|  | Afu4g09320 | dppIV | x |  |  | x |  |  | x |  |  |  |  |  |
|  | Afu4g09560 | zrfC | x |  |  | x |  |  | x |  |  | x |  |  |
|  | Afu4g11800 | alp1 |  |  |  |  |  |  |  |  |  |  |  |  |
|  | Afu4g12470 | cpcA | x | x |  | x |  |  | x |  |  | x | x |  |
|  | Afu4g13750 | mep20 |  | x |  |  |  |  |  |  |  |  |  |  |
|  | Afu5g01340 | plb2 |  | x |  | x |  |  |  |  |  |  |  |  |
|  | Afu5g03790 | fetC | x |  |  | x |  |  | x |  |  | x |  |  |
|  | Afu5g03800 | ftrA |  |  |  |  |  |  |  |  |  |  |  |  |
|  | Afu5g05480 | rhbA |  |  |  | x |  |  | x |  |  |  |  |  |
|  | Afu5g08570 | pkaC2 | x | x |  | x | x |  |  | x |  |  | x |  |
|  | Afu5g08890 | lysF | x | x |  | x | x |  |  | x |  |  | x |  |
|  | Afu5g09210 | alp2 | x |  |  |  |  |  | x |  |  | x |  |  |
|  | Afu5g11260 | sreA |  |  |  |  |  |  |  |  |  |  |  |  |
|  | Afu5g13300 | pep1 | x |  |  |  |  |  |  |  |  |  |  |  |
|  | Afu6g01970 | areA | x | x |  |  | x |  |  | x |  |  |  |  |
|  | Afu6g03590 | mcsA |  |  |  | x |  |  |  |  |  |  |  |  |
|  | Afu6g04820 | pabA | x | x |  | x | x |  |  | x |  |  | x |  |
|  | Afu6g12950 | tpsA | x |  |  | x |  |  |  |  |  |  |  |  |
|  | Afu7g04910 | Null | x | x |  | x | x |  | x | x |  | x | x |  |
|  | Afu7g04930 | pr1 |  | x |  |  | x |  |  | x |  |  |  |  |
|  | Afu7g05930 | mepB | x | x |  | x |  |  |  |  |  |  |  |  |
|  | Afu8g02760 | amcA |  |  |  |  |  |  |  |  |  |  |  |  |
|  | Afu8g07080 | mep | x | x |  | x |  |  | x | x |  |  | x |  |
| Signalling and regulation | Afu1g05800 | mkk2 | x |  |  | x |  |  | x |  |  | x |  |  |
|  | Afu1g06900 | crzA |  | x |  | x |  |  | x |  |  |  |  |  |
|  | Afu1g12930 | gpaB |  |  |  | x |  |  |  |  |  |  |  |  |
|  | Afu1g12940 | sakA |  |  |  |  |  |  |  |  |  |  |  |  |
|  | Afu1g13140 | gpaA |  |  |  |  |  |  |  |  |  |  |  |  |
|  | Afu1g15950 | pbs2 | x |  |  | x |  |  | x | x |  | x |  |  |
|  | Afu2g00660 | tcsB | x | x |  | x | x |  |  | x |  |  | x |  |
|  | Afu2g01260 | srbA |  | x |  |  | x |  |  | x |  | x | x |  |
|  | Afu2g07770 | rasB |  |  |  |  |  |  |  |  |  |  |  |  |
|  | Afu2g12200 | pkaC | x |  |  |  | x |  |  |  |  |  |  |  |
|  | Afu2g12640 | gprD | x | x |  |  | x |  | x | x |  |  |  |  |
|  | Afu2g13260 | medA | x | x |  | x | x |  |  | x |  | x | x |  |
|  | Afu3g05900 | ste7 | x | x |  | x | x |  | x |  |  | x |  |  |
|  | Afu3g10000 | pkaR |  |  |  | x |  |  | x | x |  | x |  |  |
|  | Afu3g11080 | bck1 | x | x |  | x | x |  | x | x |  | x | x |  |
|  | Afu3g11250 | ace2 | x | x |  | x | x |  | x | x |  | x | x |  |
|  | Afu4g13720 | mpkA |  |  |  |  |  |  |  |  |  | x |  |  |
|  | Afu5g06420 | steC/ste11 | x |  |  | x |  |  | x |  |  |  | x |  |
|  | Afu5g08420 | sho1 | x |  |  |  |  |  |  |  |  |  |  |  |
|  | Afu5g09100 | mpkC | x | x |  | x | x |  | x | x |  | x | x |  |
|  | Afu5g09360 | calA |  |  |  | x |  |  |  |  |  |  |  |  |
|  | Afu5g11230 | rasA |  |  |  |  |  |  |  |  |  |  |  |  |
|  | Afu5g12210 | sfaD | x |  |  |  |  |  |  |  |  |  |  |  |
|  | Afu6g08520 | acyA | x | x |  |  | x |  | x | x |  |  |  |  |
|  | Afu6g10240 | fos-1 | x | x |  | x | x |  |  | x |  | x | x |  |
|  | Afu6g12820 | mpkB | x |  |  | x |  |  | x | x |  | x |  |  |
|  | Afu7g04800 | gprC | x |  |  |  |  |  |  |  |  |  |  |  |

*null = no gene name assigned.
