# Supplementary material for: Variation of virulence of five Aspergillus fumigatus isolates in four different infection models
Source: PLoS One. 2021 Jul 9;16(7):e0252948. doi: 10.1371/journal.pone.0252948 (PMC8270121; doi:10.1371/journal.pone.0252948)
Supplement: S2 Table — (DOCX) [file pone.0252948.s006.docx]

**Supplementary Table 2.** Shared SNP’s with the location and amino acid change in *fma-PKS* (Afu8g00370) in the ATCC46645, CEA10, DTO271-B5 and DTO303-F3 strains.

| Reference (Af293) | Alternative | Location | Amino acid change |
| --- | --- | --- | --- |
| A | T | 2335 | Cysteine 🡪 Serine |
| T | C | 2082 | Asparagine 🡪 Serine |
| C | T | 1624 | Valine 🡪 Isoleucine |
| T | A | 746 | Tyrosine 🡪 Phenylalanine |
| A | G | 641 | Serine 🡪 Proline |
