# Supplementary material for: Variation of virulence of five Aspergillus fumigatus isolates in four different infection models
Source: PLoS One. 2021 Jul 9;16(7):e0252948. doi: 10.1371/journal.pone.0252948 (PMC8270121; doi:10.1371/journal.pone.0252948)
Supplement: S3 Table — Shared SNPs are displayed in bold. (DOCX) [file pone.0252948.s007.docx]

**Supplementary Table 3.** Base pair (BP) change, location and amino acid (AA) substitution in the genes with SNP’s shared between ATCC46645 and DTO303-F3. Shared SNP’s are displayed in bold.

| Gene ID | Gene name | Group | ATCC46645 | | | DTO303-F3 | | |
| --- | --- | --- | --- | --- | --- | --- | --- | --- |
|  |  |  | BP Change | Location | AA change | BP Change | Location | AA change |
| Afu6g07210 | *sod4* | Resistance to immune response | **G 🡪 A** | **30** | **Arg 🡪 Arg** | **G 🡪 A** | **30** | **Arg 🡪 Arg** |
|  |  |  |  |  |  | A 🡪 C | 54 | Thr 🡪 Pro |
|  |  |  |  |  |  | T 🡪 C | 95 | Ile 🡪 Ile |
|  |  |  |  |  |  | G 🡪 A | 115 | Leu 🡪 Leu |
|  |  |  |  |  |  | T 🡪 G | 128 | Val 🡪 Gly |
|  |  |  |  |  |  | A 🡪 C | 146 | Ser 🡪 Ser |
|  |  |  |  |  |  | A 🡪 T | 204 | Thr 🡪 Ser |
|  |  |  |  |  |  | C 🡪 A | 242 | Ala 🡪 Asp |
|  |  |  |  |  |  | A 🡪 C | 243 | His 🡪 Pro |
|  |  |  |  |  |  | A 🡪 C | 249 | Asn 🡪 His |
|  |  |  |  |  |  | A 🡪 C | 249 | Asn 🡪 Thr |
| Afu2g01870 | *chsA* | Cell wall | **G 🡪 C** | **734** | **Leu 🡪 Phe** | A 🡪 C | 906 | Val 🡪 Val |
|  |  |  | **G 🡪 T** | **645** | **Ala 🡪 Ala** | T 🡪 C | 879 | Thr 🡪 Thr |
|  |  |  | **G 🡪 A** | **442** | **Ala 🡪 Ala** | G 🡪 T | 857 | Val 🡪 Val |
|  |  |  | **C 🡪 T** | **331** | **Ala 🡪 Ala** | T 🡪 G | 780 | Lys 🡪 Asn |
|  |  |  | **A 🡪 G** | **229** | **Phe 🡪 Phe** | **G 🡪 A** | **734** | **Leu 🡪 Phe** |
|  |  |  | **CATC 🡪 GACT** | **116** | **Asp Asp 🡪 Ser His** | **G 🡪 C** | **645** | **Ala 🡪 Ala** |
|  |  |  | **T 🡪 C** | **46** | **Val 🡪 Val** | **G 🡪 A** | **442** | **Ala 🡪 Ala** |
|  |  |  | **C 🡪 T** | **33** | **Arg 🡪 Gln** | A 🡪 C | 394 | Leu 🡪 Leu |
|  |  |  | **C 🡪 A** | **22** | **Gln 🡪 His** | **C 🡪 T** | **331** | **Ala 🡪 Ala** |
|  |  |  |  |  |  | **A 🡪 G** | **229** | **Phe 🡪 Phe** |
|  |  |  |  |  |  | G 🡪 A | 192 | Pro 🡪 Ser |
|  |  |  |  |  |  | G 🡪 T | 186 | Leu 🡪 Met |
|  |  |  |  |  |  | A 🡪 C | 171 | Trp 🡪 Gly |
|  |  |  |  |  |  | T 🡪 G | 156 | Asp 🡪 Ala |
|  |  |  |  |  |  | A 🡪 C | 117 | Asp 🡪 Glu |
|  |  |  |  |  |  | **CATC 🡪 GACT** | **116** | **Asp Asp 🡪 Ser His** |
|  |  |  |  |  |  | G 🡪 T | 110 | Pro 🡪 Thr |
|  |  |  |  |  |  | T 🡪 G | 109 | Glu 🡪 Ala |
|  |  |  |  |  |  | G 🡪 T | 88 | Pro 🡪 His |
|  |  |  |  |  |  | T 🡪 G | 55 | Ser 🡪 Arg |
|  |  |  |  |  |  | **T 🡪 C** | **46** | **Val 🡪 Val** |
|  |  |  |  |  |  | **C 🡪 T** | **33** | **Arg 🡪 Gln** |
|  |  |  |  |  |  | **C 🡪 A** | **22** | **Gln 🡪 His** |
| Afu2g17970 | *fgaFS* | Toxins and secondary metabolites | **G 🡪 A** | **6** | **Leu 🡪 Leu** | **G 🡪 A** | **6** | **Leu 🡪 Leu** |
|  |  |  | **G 🡪 C** | **21** | **Asp 🡪 His** | **G 🡪 C** | **21** | **Asp 🡪 His** |
|  |  |  | **G 🡪 A** | **152** | **Lys 🡪 Lys** | C 🡪 T | 66 | Asp 🡪 Asp |
|  |  |  | **T 🡪 C** | **197** | **Gly 🡪 Gly** | T 🡪 A | 68 | Ile 🡪 Ile |
|  |  |  |  |  |  | **G 🡪 A** | **152** | **Lys 🡪 Lys** |
|  |  |  |  |  |  | **T 🡪 C** | **197** | **Gly 🡪 Gly** |
|  |  |  |  |  |  | G 🡪 C | 271 | Ser 🡪 Thr |
| Afu2g18010 | *easM* | Toxins and secondary metabolites | **C 🡪 A** | **108** | **Arg 🡪 Arg** | **C 🡪 A** | **108** | **Arg 🡪 Arg** |
|  |  |  | **G 🡪 A** | **467** | **Gln 🡪 Gln** | G 🡪 A | 114 | Ala 🡪 Ala |
|  |  |  |  |  |  | G 🡪 T | 136 | Gly 🡪 Val |
|  |  |  |  |  |  | T 🡪 C | 161 | Asp 🡪 Asp |
|  |  |  |  |  |  | T 🡪 G | 233 | Ile 🡪 Arg |
|  |  |  |  |  |  | A 🡪 C | 237 | Ile 🡪 Leu |
|  |  |  |  |  |  | A 🡪 C | 253 | Ser 🡪 Arg |
|  |  |  |  |  |  | G 🡪 T | 260 | Glu 🡪 Asp |
|  |  |  |  |  |  | A 🡪 C | 332 | Asp 🡪 Ala |
|  |  |  |  |  |  | T 🡪 G | 334 | Ile 🡪 Ser |
|  |  |  |  |  |  | T 🡪 C | 336 | Ile 🡪 Thr |
|  |  |  |  |  |  | **G 🡪 A** | **467** | **Gln 🡪 Gln** |
|  |  |  |  |  |  | G 🡪 T | 476 | Val 🡪 Phe |
|  |  |  |  |  |  | C 🡪 A | 477 | Ala 🡪 Glu |
| Afu2g18020 | *fgaAT* | Toxins and secondary metabolites | **C 🡪 G** | **364** | **Leu 🡪 Leu** | G 🡪 T | 27 | Ser 🡪 Ser |
|  |  |  |  |  |  | C 🡪 T | 51 | Ser 🡪 Phe |
|  |  |  |  |  |  | A 🡪 C | 66 | Ile 🡪 Leu |
|  |  |  |  |  |  | A 🡪 T | 69 | Lys 🡪 Asn |
|  |  |  |  |  |  | A 🡪 C | 85 | Ile 🡪 Leu |
|  |  |  |  |  |  | A 🡪 C | 184 | Asn 🡪 Thr |
|  |  |  |  |  |  | T 🡪 G | 265 | Leu 🡪 Val |
|  |  |  |  |  |  | **C 🡪 G** | **364** | **Leu 🡪 Leu** |
|  |  |  |  |  |  | G 🡪 T | 377 | Gly 🡪 Stop |
|  |  |  |  |  |  | C 🡪 T | 380 | Ala 🡪 Ala |
|  |  |  |  |  |  | A 🡪 G | 458 | Lys 🡪 Arg |
| Afu2g18030 | *fgaCAT* | Toxins and secondary metabolites | **T 🡪 C** | **97** | **Leu 🡪 Leu** | A 🡪 C | 487 | Tyr 🡪 Asp |
|  |  |  | **A 🡪 G** | **66** | **Pro 🡪 Pro** | T 🡪 C | 466 | Lys 🡪 Arg |
|  |  |  | **T 🡪 G** | **37** | **Glu 🡪 Ala** | A 🡪 C | 432 | Leu 🡪 Arg |
|  |  |  |  |  |  | C 🡪 CCGT | 391 | Asp insertion |
|  |  |  |  |  |  | T 🡪 G | 363 | Asp 🡪 Ala |
|  |  |  |  |  |  | T 🡪 G | 253 | Asp 🡪 Ala |
|  |  |  |  |  |  | T 🡪 G | 251 | Asp 🡪 Ala |
|  |  |  |  |  |  | G 🡪 A | 236 | Arg 🡪 Cys |
|  |  |  |  |  |  | A 🡪 C | 213 | Gly 🡪 Gly |
|  |  |  |  |  |  | A 🡪 C | 207 | Ser 🡪 Ala |
|  |  |  |  |  |  | A 🡪 C | 199 | Phe 🡪 Val |
|  |  |  |  |  |  | T 🡪 G | 174 | Asn 🡪 Thr |
|  |  |  |  |  |  | G 🡪 T | 169 | Arg 🡪 Arg |
|  |  |  |  |  |  | TTT 🡪 ATA | 131 | Lys 🡪 Tyr |
|  |  |  |  |  |  | C 🡪 A | 129 | Ala 🡪 Ser |
|  |  |  |  |  |  | T 🡪 A | 128 | Met 🡪 Leu |
|  |  |  |  |  |  | C 🡪 A | 127 | Gly 🡪 Trp |
|  |  |  |  |  |  | C 🡪 A | 126 | Lys 🡪 Asn |
|  |  |  |  |  |  | T 🡪 A | 126 | Lys 🡪 Stop |
|  |  |  |  |  |  | C 🡪 A | 125 | Leu 🡪 Phe |
|  |  |  |  |  |  | G 🡪 A | 124 | Asp 🡪 Asp |
|  |  |  |  |  |  | C 🡪 A | 124 | Asp 🡪 Tyr |
|  |  |  |  |  |  | C 🡪 A | 123 | Arg 🡪 Ile |
|  |  |  |  |  |  | T 🡪 A | 123 | Arg 🡪 Stop |
|  |  |  |  |  |  | C 🡪 T | 122 | Met 🡪 Ile |
|  |  |  |  |  |  | T 🡪 A | 122 | Met 🡪 Leu |
|  |  |  |  |  |  | C 🡪 A | 103 | Thr 🡪 Thr |
|  |  |  |  |  |  | **T 🡪 C** | **97** | **Leu 🡪 Leu** |
|  |  |  |  |  |  | **A 🡪 G** | **66** | **Pro 🡪 Pro** |
|  |  |  |  |  |  | A 🡪 G | 54 | Val 🡪 Ala |
|  |  |  |  |  |  | **T 🡪 G** | **37** | **Glu 🡪 Ala** |
| Afu5g12760 |  | Toxins and secondary metabolites | **T 🡪 G** | **278** | **His 🡪 Pro** | A 🡪 T | 422 | His 🡪 Gln |
|  |  |  | **C 🡪 T** | **200** | **Arg 🡪 Arg** | G 🡪 A | 410 | Arg 🡪 Cys |
|  |  |  | **C 🡪 T** | **9** | **Arg 🡪 Lys** | G 🡪 A | 359 | Ala 🡪 Val |
|  |  |  |  |  |  | A 🡪 C | 358 | Cys 🡪 Trp |
|  |  |  |  |  |  | T 🡪 G | 292 | Thr 🡪 Pro |
|  |  |  |  |  |  | T 🡪 G | 282 | Tyr 🡪 Ser |
|  |  |  |  |  |  | **T 🡪 G** | **278** | **His 🡪 Pro** |
|  |  |  |  |  |  | A 🡪 C | 250 | Asn 🡪 Lys |
|  |  |  |  |  |  | T 🡪 G | 228 | Lys 🡪 Asn |
|  |  |  |  |  |  | **C 🡪 T** | **200** | **Arg 🡪 Arg** |
|  |  |  |  |  |  | G 🡪 A | 114 | His 🡪 Tyr |
|  |  |  |  |  |  | TA 🡪 AT | 86 | Met 🡪 Leu |
|  |  |  |  |  |  | T 🡪 C | 35 | Asn 🡪 Asp |
|  |  |  |  |  |  | T 🡪 C | 33 | Ser 🡪 Gly |
|  |  |  |  |  |  | C 🡪 T | 31 | Ala 🡪 Thr |
|  |  |  |  |  |  | **C 🡪 T** | **9** | **Arg 🡪 Lys** |
| Afu3g00590 | *aspHS* | Allergens | **A 🡪 G** | **51** | **Val 🡪 Val** | T 🡪 G | 122 | Asn 🡪 Thr |
|  |  |  | **G 🡪 T** | **27** | **Gln 🡪 Lys** | A 🡪 C | 116 | Val 🡪 Gly |
|  |  |  |  |  |  | **A 🡪 G** | **51** | **Val 🡪 Val** |
|  |  |  |  |  |  | **G 🡪 T** | **27** | **Gln 🡪 Lys** |
