# Supplementary material for: Variation of virulence of five Aspergillus fumigatus isolates in four different infection models
Source: PLoS One. 2021 Jul 9;16(7):e0252948. doi: 10.1371/journal.pone.0252948 (PMC8270121; doi:10.1371/journal.pone.0252948)
Supplement: S4 Table — (DOCX) [file pone.0252948.s008.docx]

**Supplementary Table 4**. Strains used for the assembly of the phylogenetic tree.

| Strain ID | Reference |
| --- | --- |
| IFM 59355-1 | [2] |
| IFM 59355-1 | [2] |
| IFM 59355-2 | [2] |
| IFM 59355-2 | [2] |
| IFM 59356-1 | [2] |
| IFM 59356-1 | [2] |
| IFM 59356-2 | [2] |
| IFM 59356-2 | [2] |
| IFM 59356-3 | [2] |
| IFM 59356-3 | [2] |
| IFM 59361-1 | [2] |
| IFM 59361-2 | [2] |
| IFM 60237 | [2] |
| Af293 | [3] |
| Af65 | [3] |
| 12-7505446 | [3] |
| 12-7505220 | [3] |
| 09-7500806 | [3] |
| 12-7504652 | [3] |
| 12-7504462 | [3] |
| 12-7505054 | [3] |
| 08-12-12-13 | [3] |
| 08-36-03-25 | [3] |
| 08-31-08-91 | [3] |
| 08-19-02-61 | [3] |
| 08-19-02-30 | [3] |
| 10-01-02-27 | [3] |
| 08-19-02-46 | [3] |
| 08-19-02-10 | [3] |
| Afu_942/09 | [3] |
| Afu_1042/09 | [3] |
| Afu_343/P/11 | [3] |
| Afu_591/12 | [3] |
| Afu_124/E11 | [3] |
| Afu_166/E11 | [3] |
| Afu_257/E11 | [3] |
| Afu_218/E11 | [3] |
| A1163 | [4] |
| LMB-35Aa | [5] |
| ISSF_21 | [6] |
| 1F1SW_F4 | [6] |
| MO68507EXP | [7] |
| MO54056EXP | [7] |
| MO76959EXP | [7] |
| MO69250EXP | [7] |
| MO79587EXP | [7] |
| MO78722EXP | [7] |
| MO91298SB | [7] |
| MO89263LAB | [7] |
| V130-54 | [8] |
| V130-18 | [8] |
| V130-14 | [8] |
| V130-15 | [8] |
| V157-48 | [8] |
| V157-40 | [8] |
| V157-39 | [8] |
| V157-59 | [8] |
| V157-47 | [8] |
| V157-62 | [8] |
| V157-61 | [8] |
| V157-60 | [8] |
| V157-80 | [8] |
