# Supplementary material for: Variation of virulence of five Aspergillus fumigatus isolates in four different infection models
Source: PLoS One. 2021 Jul 9;16(7):e0252948. doi: 10.1371/journal.pone.0252948 (PMC8270121; doi:10.1371/journal.pone.0252948)
Supplement: S5 Table — Table is based upon data in the figures of the main in the main paper, used statistics can be found there and in the material and methods section. (DOCX) [file pone.0252948.s009.docx]

**Supplementary Table 5.** Summary of comparison of *A. fumigatus* strains. Table is based upon data in the figures of the main in the main paper, used statistics can be found there and in the material and methods section.

|  | **Af293** | **ATCC46645** | **CEA10** | **DTO271-B5** | **DTO303-F3** |
| --- | --- | --- | --- | --- | --- |
| ***Stress*** |  |  |  |  |  |
| Peroxide (mm) | 32 | 26 | 27 | 29 | 26 |
| Menadione (mm) | 37 | 27 | 29 | 29 | 28 |
|  |  |  |  |  |  |
| ***A549*** | **Af293** | **ATCC46645** | **CEA10** | **DTO271-B5** | **DTO303-F3** |
| Association (conidia/cell) | 0.19 | 0.14 | 0.17 | 0.11 | 0.12 |
| Internalization (%) | 82 | 77 | 77 | 77 | 80 |
| Germination (%) | 68 | 87 | 75 | 86 | 85 |
| IL-8 release 12 h | 8.2 | 18.2 | 6.6 | 7.8 | 8.8 |
| LDH release 12 h | 8.1 | 8.4 | 6.2 | 10.1 | 14.6 |
| Apoptotic/necrotic cells 12 h | *Similar for all strains* | | | | |
|  | | | | | |
| ***Protostelium aurantium*** | **Af293** | **ATCC46645** | **CEA10** | **DTO271-B5** | **DTO303-F3** |
| Uptake (4.5h swollen) (%) | 16 | 35 | 30 | 28 | 32 |
| Fungal survival (%) |  |  |  |  |  |
| 4 h swollen | 88 | 56 | 67 | 60 | 52 |
| 6 h swollen | 62 | 47 | 28 | 36 | 28 |
| 8 h swollen | 71 | 96 | 127 | 90 | 80 |
| Germination with amoeba (% hyphae) |  |  |  |  |  |
| 4 h swollen | 0 | 0.5 | 0 | 11 | 2 |
| 6 h swollen | 1 | 16 | 2 | 47 | 29 |
| 8 h swollen | 0.8 | 42 | 0 | 49 | 63 |
| Germination no amoeba (% germlings) |  |  |  |  |  |
| 4 h swollen | 0 | 0 | 0.2 | 0.4 | 0.8 |
| 6 h swollen | 0.8 | 10 | 3 | 2 | 4 |
| 8 h swollen | 3 | 54 | 39 | 20 | 25 |
| Survival of amoeba (%) |  |  |  |  |  |
| 4 h swollen | 48 | 31 | 50 | 69 | 39 |
| 6 h swollen | 48 | 29 | 49 | 65 | 44 |
| 8 h swollen | 97 | 46 | 106 | 97 | 55 |
|  | | | | | |
| ***Galleria melonella*** | **Af293** | **ATCC46645** | **CEA10** | **DTO271-B5** | **DTO303-F3** |
| Survival *Galleria melonella* (%) |  |  |  |  |  |
| 1*10^5^ conidia mL^-1^ | 87 | 73 | 73 | 53 | 33 |
| 1*10^6^ conidia mL^-1^ | 80 | 47 | 40 | 40 | 13 |
| 1*10^7^ conidia mL^-1^ | 60 | 20 | 53 | 33 | 27 |
| Survival conidia | *Similar for all strains* | | | | |
|  | | | | | |
| ***Zebrafish*** | **Af293** | **ATCC46645** | **CEA10** | **DTO271-B5** | **DTO303-F3** |
| Survival zebrafish embryo’s (%) | 92 | 82 | 66 | 79 | 79 |
