# Supplementary material for: Variation of virulence of five Aspergillus fumigatus isolates in four different infection models
Source: PLoS One. 2021 Jul 9;16(7):e0252948. doi: 10.1371/journal.pone.0252948 (PMC8270121; doi:10.1371/journal.pone.0252948)
Supplement: S6 Table — The SNPs described by [58] is highlighted in bold. (DOCX) [file pone.0252948.s010.docx]

**Supplementary Table 6.** Base pair (BP) change, location and amino acid (AA) substitution in the *FtmD* (Afu8g00200) gene. The SNPs described by (9] is highlighted in bold.

| BP change | Location | AA change | Strain | | | |
| --- | --- | --- | --- | --- | --- | --- |
|  |  |  | ATCC46645 | CEA10 | DTO271-B5 | DTO303-F3 |
| A 🡪 C | 14 | Ile 🡪 Leu |  |  | X | X |
| A 🡪 C | 20 | Asp 🡪 Ala |  |  | X | X |
| C 🡪 T | 75 | Ile 🡪 Ile |  |  | X | X |
| G 🡪 A | 83 | Ala 🡪 Thr |  |  | X | X |
| A 🡪 C | 109 | Ser 🡪 Arg |  |  | X | X |
| A 🡪 C | 132 | Thr 🡪 Pro |  |  | X | X |
| C 🡪 T | 141 | Pro 🡪 Leu |  |  | X | X |
| A 🡪 C | 145 | Lys 🡪 Thr |  |  | X | X |
| T 🡪 C | 160 | Arg 🡪 Arg | X | X | X | X |
| T 🡪 G | 175 | Val 🡪 Gly |  |  | X | X |
| **T 🡪 G** | **202** | **Leu 🡪 Arg** | **X** | **X** | **X** | **X** |
| T 🡪 C | 233 | Ile 🡪 Thr | X | X | X | X |
| C 🡪 T | 249 | Ala 🡪 Val |  |  | X | X |
| A 🡪 C | 271 | Met 🡪 Leu |  |  | X | X |
| T 🡪 G | 299 | Leu 🡪 Arg |  |  | X | X |
| C 🡪 T | 311 | Arg 🡪 Arg |  |  | X | X |
| T 🡪 G | 314 | Val 🡪 Gly |  |  | X | X |
