# Supplementary material for: Variation of virulence of five Aspergillus fumigatus isolates in four different infection models
Source: PLoS One. 2021 Jul 9;16(7):e0252948. doi: 10.1371/journal.pone.0252948 (PMC8270121; doi:10.1371/journal.pone.0252948)
Supplement: S1 File — (DOCX) [file pone.0252948.s011.docx]

**References**

[1] Puértolas-Balint F, Rossen JWA, Oliveira Dos Santos C, Chlebowicz MMA, Raangs EC, van Putten ML, et al. Revealing the Virulence Potential of Clinical and Environmental Aspergillus fumigatus Isolates Using Whole-Genome Sequencing. Front Microbiol 2019;10:1970.

[2] Hagiwara D, Takahashi H, Watanabe A, Takahashi-Nakaguchi A, Kawamoto S, Kamei K, et al. Whole-genome comparison of Aspergillus fumigatus strains serially isolated from patients with aspergillosis. J Clin Microbiol 2014 Dec;52(12):4202-4209.

[3] Abdolrasouli A, Rhodes J, Beale MA, Hagen F, Rogers TR, Chowdhary A, et al. Genomic Context of Azole Resistance Mutations in Aspergillus fumigatus Determined Using Whole-Genome Sequencing. mBio 2015 Jun 02,;6(3):e00536.

[4] Fedorova ND, Khaldi N, Joardar VS, Maiti R, Amedeo P, Anderson MJ, et al. Genomic islands in the pathogenic filamentous fungus Aspergillus fumigatus. PLoS Genet 2008 Apr 11,;4(4):e1000046.

[5] Paul S, Zhang A, Ludeña Y, Villena GK, Yu F, Sherman DH, et al. Insights from the genome of a high alkaline cellulase producing Aspergillus fumigatus strain obtained from Peruvian Amazon rainforest. J Biotechnol 2017 Jun 10,;251:53-58.

[6] Singh NK, Blachowicz A, Checinska A, Wang C, Venkateswaran K. Draft Genome Sequences of Two Aspergillus fumigatus Strains, Isolated from the International Space Station. Genome Announc 2016 -7-14;4(4).

[7] Lind AL, Wisecaver JH, Lameiras C, Wiemann P, Palmer JM, Keller NP, et al. Drivers of genetic diversity in secondary metabolic gene clusters within a fungal species. PLoS Biol 2017 Nov;15(11):e2003583.

[8] Ballard E, Melchers WJG, Zoll J, Brown AJP, Verweij PE, Warris A. In-host microevolution of Aspergillus fumigatus: A phenotypic and genotypic analysis. Fungal Genet Biol 2018 04;113:1-13.

[9] Kato N, Suzuki H, Okumura H, Takahashi S, Osada H. A point mutation in ftmD blocks the fumitremorgin biosynthetic pathway in Aspergillus fumigatus strain Af293. Biosci Biotechnol Biochem 2013;77(5):1061-1067.
